# Experiences and support needs of patients receiving home mechanical ventilation and their caregivers: a qualitative meta-synthesis

**DOI:** 10.3389/fpubh.2026.1793552

**Published:** 2026-07-02

**Authors:** Chengxiang Fan, Yanjie Ma, Xiling Shao, Huili Pei, Jinpei Gong, Chengli Guo, Xiaoxia Guo, Yifan Zhang, Juan Wang

**Affiliations:** 1 School of Nursing, Henan Medical College, Zhengzhou, China; 2 Wuxi School of Medicine, Jiangnan University, Wuxi, China; 3 Department of Neurosurgery, Henan Provincial People's Hospital, Zhengzhou, China; 4 Shi’s Center of Orthopedics and Traumatology, Shuguang Hospital Affiliated to Shanghai University of Traditional Chinese Medicine, Shanghai, China; 5 School of Nursing, Beihua University, Jilin, China; 6 School of Nursing, Shanghai Donghai College, Shanghai, China

**Keywords:** caregiver experience, home mechanical ventilation, meta-synthesis, patient experience, qualitative research, support needs, systematic review

## Abstract

**Background:**

Home mechanical ventilation (HMV) is an important form of support for the out-of-hospital management of patients with chronic respiratory failure and long-term ventilatory dependence. It plays an important role in prolonging life, relieving symptoms, and supporting home living. As the site of treatment extends from the hospital to the home, patients and caregivers must jointly face challenges related to treatment decision-making, technological adaptation, expansion of caregiving responsibilities, and insufficient support systems. Existing studies have often focused on a single disease, a single ventilation modality, or a single caregiving perspective, and a systematic synthesis of the experiences and support needs of patients receiving HMV and their caregivers remains lacking.

**Objective:**

This study aimed to systematically synthesize the experiences and support needs of patients receiving HMV and their caregivers during home treatment through a qualitative systematic review and meta-synthesis. It also explored core issues across different disease types, ventilation modalities, levels of dependence, and caregiving contexts to provide evidence for developing stratified and continuous care support pathways for the family as a unit.

**Methods:**

This study conducted a meta-synthesis using the Joanna Briggs Institute methodology for qualitative systematic reviews. PubMed, Web of Science, Embase, the Cochrane Library, and CINAHL were systematically searched from January 1, 2020, to April 30, 2026, and the reference lists of the included studies were manually searched. The methodological quality of the included studies was assessed using the JBI Critical Appraisal Checklist for Qualitative Research. Findings were categorized and synthesized using the JBI meta-aggregation approach, and confidence in the evidence for the synthesized themes was assessed using the ConQual framework.

**Results:**

Thirteen qualitative studies were included, involving 129 patients receiving HMV and 121 family-caregiving-related participants, including family caregivers, relatives, and bereaved family members. Five synthesized themes and 17 subthemes were generated: (1) passive entry, repeated weighing, and active participation in HMV decision-making; (2) adapting to the integration of ventilation technology into everyday family life; (3) ongoing tensions among life support, quality of life, and autonomy; (4) expansion of family caregiving responsibilities and reconstruction of the boundaries of professional care; and (5) gaps in support systems and the need for continuous support for the whole family. The ConQual assessment showed that the final level of confidence was moderate for all five synthesized themes.

**Conclusion:**

HMV is not only a long-term respiratory support technology but also a continuous care process deeply embedded in disease progression, family life, and healthcare service systems. The experiences of patients and caregivers are jointly influenced by disease type, ventilation modality, level of ventilatory dependence, socioeconomic conditions, and healthcare system context. HMV nursing practice should shift from individual patient management and guidance on device use toward continuous support for the family as a unit. Particular attention should be given to strengthening shared decision-making, caregiver training, professional follow-up, remote monitoring and digital follow-up, psychosocial support, resource navigation, and integration of early palliative care to improve the sense of security, quality of life, and sustainability of care for both patients and caregivers.

## Graphical abstract

**Figure:**
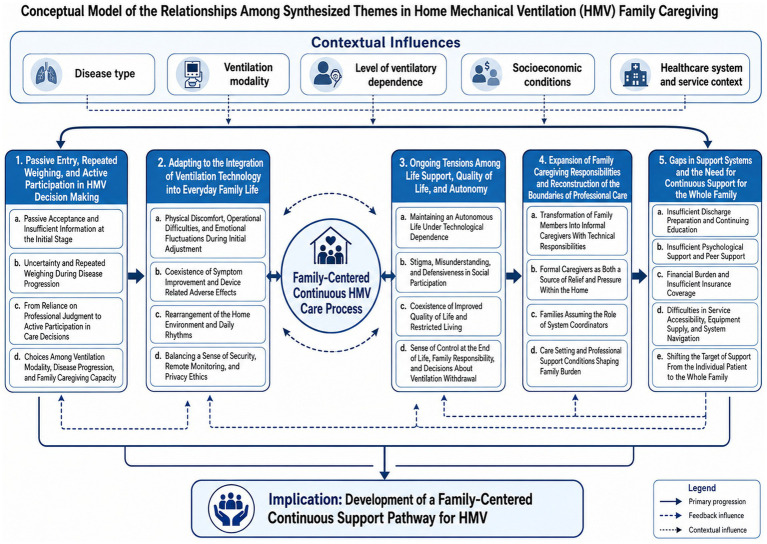

## Introduction

With advances in critical care medicine, respiratory therapy technologies, and home-based care services, the site of care for patients with chronic respiratory failure and long-term ventilatory dependence has gradually expanded from hospitals to homes and community settings (1, 2). Home mechanical ventilation (HMV) generally refers to the long-term provision of mechanical ventilatory support outside the hospital, including home and community settings, for patients with chronic respiratory failure, long-term ventilatory dependence, or impaired respiratory pump function (1, 3). Its principal modalities include non-invasive ventilation (NIV) delivered via non-invasive interfaces such as masks, for example bilevel positive airway pressure, and invasive home mechanical ventilation (IHMV) delivered through a tracheostomy (3, 4). HMV is commonly used in populations with neuromuscular diseases, amyotrophic lateral sclerosis/motor neuron disease, chronic obstructive pulmonary disease, obesity hypoventilation syndrome, chronic hypercapnia, and other conditions leading to long-term respiratory insufficiency. The degree of ventilatory dependence may range from nocturnal use and partial daytime use to near-continuous dependence (3–6). Some patients may also receive oxygen therapy; however, oxygen therapy alone does not constitute HMV. Similarly, patients receiving continuous positive airway pressure therapy solely for uncomplicated obstructive sleep apnea or obesity-related obstructive sleep apnea should not be conflated with patients who have chronic respiratory failure or long-term ventilatory dependence (3, 4, 7).

HMV plays an important role in prolonging life, alleviating dyspnea, improving sleep quality, and reducing dependence on hospital-based care, and has gradually become an important component of long-term management for patients with chronic respiratory failure (8, 9). Unlike in-hospital mechanical ventilation, HMV places complex medical technology within the context of everyday family life, enabling patients to maintain some degree of daily activity and social participation in a familiar environment (10). However, this shift also means that responsibility for care, risk monitoring, and equipment management is partly transferred from professional healthcare institutions to the home (11). Patients must not only adapt to long-term technological dependence, physical discomfort, altered daily routines, and restricted autonomy, but may also face complex issues related to disease progression, transition between ventilation modalities, end-of-life decision-making, and uncertainty about future illness and care trajectories (10–12). In amyotrophic lateral sclerosis/motor neuron disease and other progressive neuromuscular disorders in particular, HMV is not only a form of symptom management but is also closely linked to life prolongation, decisions about invasive ventilation, withdrawal of ventilation, and advance care planning (6, 12). By contrast, the experiences of patients receiving long-term NIV for conditions such as chronic obstructive pulmonary disease, obesity hypoventilation syndrome, and chronic hypercapnia may be more closely centered on adaptation to ventilation, mask tolerance, sleep improvement, and sustained adherence (5, 8, 9). These considerations indicate that the experiences of patients receiving HMV do not follow a single uniform pattern, but are jointly shaped by disease type, ventilation modality, level of dependence, and stage in the illness trajectory (10, 12).

Family caregivers play a crucial yet often underestimated role in HMV care. Whether patients can receive ventilation safely and consistently at home depends, to a large extent, on caregivers’ continuous involvement in equipment maintenance, symptom observation, alarm management, airway care, nocturnal monitoring, and emergency response (13). For some patients who are highly dependent on ventilation or receive IHMV, the responsibilities of family caregivers extend far beyond general assistance with daily living and constitute a form of informal yet technically complex care (14). During long-term caregiving, caregivers experience not only physical exhaustion and sleep deprivation, but also sustained psychological burden arising from inadequate professional training, limited opportunities for respite, financial strain, and the pressure of coordinating services (15). The involvement of formal caregivers, home nurses, and personal assistants may alleviate caregiving burden to some extent, but it may also reshape domestic space, intimate relationships, and role boundaries, turning the home into both a living environment and a site of medical care (16). Therefore, support needs related to HMV should be considered not only at the level of the individual patient but also within the broader family caregiving system.

Previous studies have examined the experiences of patients receiving HMV and those of caregivers separately, revealing patients’ complex experiences related to technological adaptation, maintenance of autonomy, changes in quality of life, and end-of-life decision-making. These studies have also indicated that caregivers have substantial support needs in relation to undertaking technically demanding care tasks, coordinating services, and managing emotional stress (17, 18). However, the existing evidence remains fragmented. First, many studies have focused on a single disease, a single ventilation modality, or a single caregiving perspective, making it difficult to capture differences in experiences across disease contexts, levels of ventilatory dependence, and healthcare systems (19, 20). Second, some studies have discussed HMV together with long-term oxygen therapy, continuous positive airway pressure therapy for uncomplicated obstructive sleep apnea, or other forms of home respiratory therapy, which may obscure the boundaries of the target population (7, 19, 21). In addition, factors such as socioeconomic status, insurance coverage, equipment supply, community services, and access to professional support may substantially shape the experiences of patients and caregivers, but these factors have not been adequately addressed in the existing literature (15, 22–24). The experiences of patients and caregivers are not independent of each other; rather, they interact within the family caregiving context and jointly shape the long-term adaptation process associated with HMV (24). Without systematic integration of these dual perspectives, it is difficult for clinical practice to develop a genuinely family-centered pathway for continuous support.

Therefore, this study used a qualitative meta-synthesis approach (25) to systematically integrate the experiences and support needs of patients receiving HMV and their caregivers during home-based treatment. This approach enables empirical findings from different studies to be synthesized while preserving the contextual features of the original research, thereby providing a more systematic understanding of the family caregiving process in HMV. On the basis of a clear definition of HMV and explicit inclusion boundaries, this study examined how experiences were shaped by disease type, ventilation modality, level of dependence, socioeconomic conditions, and healthcare support contexts. It also compared the commonalities and differences between patients and caregivers in relation to decision-making, adaptation, caregiving responsibilities, and support needs. The aim of this study was to identify the core challenges in HMV family caregiving, the experiential patterns through which patients and caregivers influence each other, and unmet support needs, thereby providing evidence to inform the design of nursing interventions, improve discharge transition and follow-up support, and develop a family-centered model of continuous care.

## Methods

### Study design

This study used a qualitative meta-synthesis approach to systematically integrate the experiences and support needs of patients receiving HMV and their caregivers during home-based treatment. The study followed the Joanna Briggs Institute (JBI) methodology for qualitative evidence synthesis and its reporting guidance to ensure that evidence generation was systematic, transparent, and traceable (25, 26). This study focused on the experiences, adaptation processes, participation in decision-making, and support needs of patients and caregivers during long-term mechanical ventilation in home or community settings, rather than evaluating the clinical effectiveness of HMV.

### Search strategy

A systematic search was conducted in PubMed, Web of Science, Embase, the Cochrane Library, and CINAHL for studies published from January 1, 2020, to April 30, 2026. To identify relevant studies as comprehensively as possible, the reference lists of the included studies were also searched manually. The PubMed search structure and search terms are presented in Table 1. Search terms were developed around the core concepts of “home mechanical ventilation,” “domiciliary ventilation,” “non-invasive ventilation,” “tracheostomy ventilation,” “patient experience,” “caregiver experience,” and “qualitative research,” and were adapted according to the controlled vocabulary and free-text search requirements of each database. The complete search strategies for all databases are provided in Appendix 1.

**Table 1:** Search terms and structure in PubMed.

Search no.	Search terms and structure
#1	“home mechanical ventilation” OR “domiciliary mechanical ventilation” OR “home ventilation” OR “long-term mechanical ventilation” OR “prolonged mechanical ventilation”
#2	“home care” OR “home setting” OR “community setting” OR domiciliary OR home
#3	“mechanical ventilation” OR “ventilation, mechanical” OR “noninvasive ventilation” OR “non-invasive ventilation” OR NIV OR BiPAP OR “bilevel positive airway pressure” OR “tracheostomy ventilation”
#4	#1 OR (#2 AND #3)
#5	attitudes OR perceptions OR perspective OR perspectives OR experience OR experiences OR feelings OR needs OR demand OR support OR “support needs” OR barriers OR facilitators
#6	qualitative OR “focus group*” OR interview* OR experienc* OR attitud* OR feel* OR respons* OR perspectiv* OR opin* OR phenomenolog* OR “lived experience*” OR narrative* OR ethnograph* OR “grounded theory” OR “content analysis”
#7	#4 AND #5 AND #6
#8	#7 AND 2020/01/01:2026/04/30[dp]

### Definition and inclusion boundaries of home mechanical ventilation

In this study, home mechanical ventilation was defined as long-term mechanical ventilatory support provided to patients outside hospital settings, in home or community settings. It mainly included long-term NIV delivered through non-invasive interfaces such as masks and IHMV delivered through a tracheostomy (3, 4). Eligible participants could include individuals receiving nocturnal ventilation, partial daytime ventilation, or near-continuous ventilation, with the specific level of ventilatory dependence determined according to the reports of the original studies. Because definitions of long-term HMV may vary across disease types, ventilation modalities, and national or regional care systems, this study did not prespecify a uniform minimum duration of HMV use. Instead, eligibility was judged according to how the original studies defined long-term, home-based, or community-based continuous mechanical ventilatory support (3, 4). During data extraction and interpretation of findings, this study also considered the disease type, ventilation modality, duration of use or level of dependence, and caregiving context reported in the original studies.

To avoid conceptual ambiguity, this study did not regard long-term oxygen therapy alone as HMV. Patients receiving oxygen therapy alone, short-term acute mechanical ventilation, in-hospital or ICU mechanical ventilation, or CPAP therapy solely for uncomplicated obstructive sleep apnea or obesity-related obstructive sleep apnea were not included. Mixed studies involving both HMV and non-HMV populations or intervention forms were included in the synthesis only when HMV-related qualitative data could be clearly identified or extracted separately. If such data could not be distinguished, the study was excluded.

### Inclusion and exclusion criteria

The inclusion and exclusion criteria were developed according to the PICoS framework (27), as detailed in Table 2. During criteria development, the research team further clarified the operational definition of HMV and its distinction from oxygen therapy alone, short-term acute mechanical ventilation, and CPAP therapy for uncomplicated OSA to improve the transparency and consistency of participant definition.

**Table 2:** Inclusion and exclusion criteria.

PICoS element	Inclusion criteria	Exclusion criteria
Population	Adults aged ≥18 years receiving HMV in home or community settings, including NIV delivered through a mask or other non-invasive interface, such as BiPAP or bilevel positive airway pressure ventilation, and invasive ventilation delivered via tracheostomy. Informal caregivers, including family members, spouses, relatives, or other unpaid caregivers, were also included	Individuals not receiving HMV; patients receiving oxygen therapy alone; patients receiving short-term acute mechanical ventilation in hospital or ICU settings; patients using CPAP solely for uncomplicated obstructive sleep apnea or obesity-related obstructive sleep apnea; participants aged <18 years; studies focusing exclusively on healthcare professionals or formal paid caregivers; animal studies or non-human subjects
Phenomenon of interest	Patients’ or informal caregivers’ experiences, perceptions, attitudes, emotional responses, decision-making processes, support needs, barriers, or facilitators related to HMV.	Studies not focusing on experiences or support needs; studies reporting only clinical outcomes, adherence rates, physiological indicators, complications, or device performance.
Context	Real-world home or community settings where HMV was delivered as part of long-term care. Studies involving nocturnal, intermittent daytime, or near-continuous ventilation were eligible if ventilation was delivered outside acute hospital settings	Studies conducted solely in hospital, ICU, emergency, perioperative, simulated, or experimental settings; studies unrelated to long-term home ventilatory support
Study design	Qualitative studies and mixed-methods studies with clearly extractable qualitative findings	Quantitative studies without qualitative components; reviews, editorials, protocols, conference abstracts; mixed-methods studies without separately extractable qualitative data; studies without full-text availability; studies rated as low methodological quality
Language and publication type	Full-text articles published in English or Chinese	Studies published in other languages; abstracts, posters, or records without sufficient qualitative data

### Study selection

Search results were imported into EndNote 21 for the removal of duplicates. Two researchers trained in systematic review methods then independently conducted two rounds of screening. The first round was based on titles and abstracts, and the second involved full-text review to determine final eligibility. Disagreements were first resolved through discussion. If consensus could not be reached, a third researcher reviewed the record and made the final decision. The study selection process was presented using a PRISMA flow diagram.

### Methodological quality appraisal

The methodological quality of the included studies was independently assessed and recorded using the JBI Critical Appraisal Checklist for Qualitative Research. This tool contains 10 items, which assess the congruity between the stated philosophical perspective and the research methodology, the congruity between the research methodology and the research question, the appropriateness of data collection and data analysis methods, researcher positioning and reflexivity, representation of participants’ voices, ethical approval, and the congruity between the conclusions and the interpretation of data. Each item was rated as “yes,” “no,” “unclear,” or “not applicable”.

To facilitate presentation of the overall methodological quality of the included studies, the overall quality of each study was classified as Level A, Level B, or Level C based on the completion of the JBI appraisal items and the approach used in previous qualitative systematic reviews. Level A indicated high methodological quality, Level B indicated moderate methodological quality, and Level C indicated low methodological quality. Only studies rated as Level A or Level B were included in the synthesis. Quality appraisal was independently conducted by two researchers. Disagreements were first resolved through discussion. If consensus could not be reached, a third researcher reviewed the item and made the final decision.

### Data extraction

After reading the full texts, two researchers independently extracted data using a predefined standardized extraction form. The extracted information included the first author and year of publication, country, participants and sample size, study aim and design, data collection methods, interview duration, data analysis methods, and main reported findings.

To support the interpretation of experiential differences that may arise from different study contexts, this study further recorded, when available in the original reports, disease type, ventilation modality, duration of HMV use or level of dependence, types of patients and caregivers, national or healthcare system context, and information related to socioeconomic conditions or healthcare support. For studies involving mixed populations, only qualitative data that could be clearly attributed to HMV users, HMV caregivers, or HMV-related caregiving contexts were extracted.

After completing data extraction, the two researchers compared their results. Any inconsistencies were first resolved through discussion. If consensus could not be reached, a third researcher reviewed the relevant information and made the final decision. The verified extraction results were used to describe the characteristics of the included studies and to inform the subsequent evidence synthesis.

### Meta-synthesis

This study followed the JBI methodology for qualitative evidence synthesis and used a meta-aggregation approach to systematically synthesize existing qualitative evidence on the experiences and support needs of patients receiving HMV and their caregivers during home-based treatment. Two researchers repeatedly read all included studies, extracted findings explicitly reported by the authors of the original studies, and assessed the extent to which each finding was supported by the original data by considering participants’ direct quotations. Findings with similar meanings were then grouped into categories, which were further developed into synthesized findings.

During the synthesis process, attention was also paid to the commonalities and differences between patient and caregiver perspectives, as well as the potential influence of disease type, ventilation modality, caregiving context, and support conditions on their experiences. Discrepancies in categorization or interpretation were resolved through discussion within the research team and were reviewed by a third researcher when necessary. The research team retained an analytical audit trail linking the findings of the original studies to categories and synthesized findings to enhance the transparency and traceability of the synthesis process.

### ConQual assessment of the synthesized findings

This study assessed confidence in the evidence for the final synthesized themes using the ConQual framework, Confidence in the Evidence from Reviews of Qualitative Research, which is recommended by the Joanna Briggs Institute (JBI) for qualitative evidence synthesis (28). ConQual assessment starts from a high level of confidence and applies downgrading judgments according to two core dimensions: the methodological dependability of the included studies and the credibility of the research findings. The final level of confidence is classified as high, moderate, low, or very low.

Methodological dependability was primarily assessed on the basis of five key items in the JBI Critical Appraisal Checklist for Qualitative Research. These items mainly reflect methodological congruity in the included studies, the appropriateness of data collection and analysis, the appropriateness of interpretation, and the congruity between conclusions and data interpretation. If the relevant items were generally well addressed, no downgrading was applied. If methodological limitations were identified, downgrading was conducted according to the extent to which these limitations affected the synthesized theme.

The assessment of credibility focused on the extent to which research findings were supported by participants’ original statements. According to the ConQual framework, research findings can be categorized as unequivocal, credible, or unsupported. If a synthesized theme was mainly composed of unequivocal findings, no downgrading was applied. If it contained a substantial proportion of credible findings, downgrading was considered according to the extent to which they affected confidence in the synthesized theme. If findings lacked support from participants’ original data, the confidence level was further downgraded. The final ConQual rating was determined jointly by downgrading decisions related to methodological dependability and credibility.

By applying ConQual assessment to each synthesized theme, this study further presented the strength and confidence of the qualitative evidence, helping readers understand the evidential basis of the synthesized findings.

### Ethics

This study was a systematic review and meta-synthesis and did not involve the collection of primary data; therefore, ethical approval was not required. All included studies reported ethical approval and participant informed consent.

## Results

### Study selection

**Figure 1:**
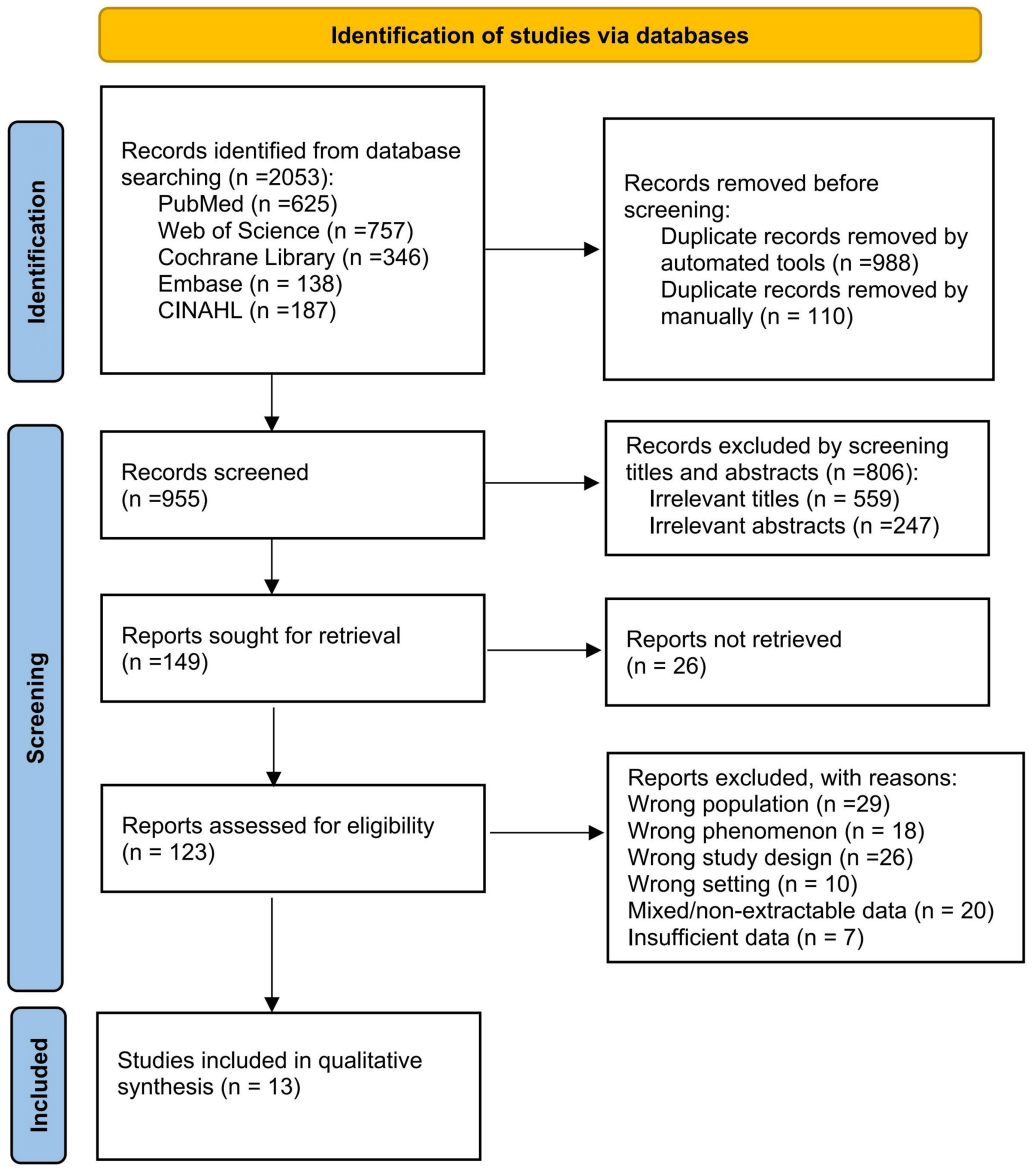
Search flow diagram.

A total of 2053 records were identified through the initial database search. After duplicate removal using EndNote 21, titles and abstracts were screened, and 123 articles underwent full-text review. Finally, 13 qualitative studies (11, 15, 23, 29–38) met the inclusion criteria and were included in this review, as shown in Figure 1. The included studies were conducted in the United Kingdom (*n* = 3), Iran (*n* = 2), Germany (*n* = 2), Denmark (*n* = 2), Sweden (*n* = 1), New Zealand (*n* = 1), Canada (*n* = 1), and Singapore (*n* = 1).

This review involved a total of 129 patients receiving HMV, including long-term NIV and IHMV were reported, as well as 121 family-caregiving-related participants. Among these family-caregiving-related participants, 85 were current family caregivers or family members, and 36 were bereaved family members. Although bereaved family members were not current caregivers actively undertaking caregiving responsibilities, their previous caregiving experiences could reflect family support needs and long-term impacts during the process of home mechanical ventilation care. Therefore, they were described within the overall group of family-caregiving-related participants. Participants such as healthcare professionals, home nurses, care assistants, and peer supporters were not included in the count of patients and family-caregiving-related participants.

The included studies mainly involved contexts of long-term NIV and IHMV delivered through a tracheostomy. For individual studies that involved both long-term oxygen therapy and home mechanical ventilation, this review only counted participants and extracted data that could be clearly attributed to HMV-related contexts. The basic characteristics of the included studies are presented in Table 3, and the complete study characteristics are provided in Appendix 2.

**Table 3:** Brief data from the studies selected for the review.

Author, year	Country	Participants and HMV context	Study design	Data collection and analysis	Main focus
Choyce et al. 2025 (29)	UK	9 adults with cystic fibrosis using long-term domiciliary NIV	Descriptive qualitative study	In-depth semi-structured interviews; thematic analysis	Lived experiences of long-term domiciliary NIV, including symptom relief, treatment acceptance, and device-related challenges
Esmaeili et al. 2022 (15)	Iran	9 family caregivers, 3 home nurses, and 3 home care attendants caring for adults receiving invasive HMV	Qualitative study using conventional content analysis	Semi-structured interviews, structured observations, and field notes; conventional content analysis	Family caregivers’ educational, psychological, economic, and transition-related needs in invasive HMV care
Ewers et al. 2022 (11)	Germany	27 patients receiving HMV and 9 relatives	Explorative qualitative cross-sectional study	Semi-structured interviews; qualitative content analysis	Experiences of medical technical aid supply, technology adaptation, risk management, and collaboration with care providers
Israelsson-Skogsberg et al. 2024 (30)	Sweden	9 young adults using HMV, including NIV, invasive ventilation, and CPAP via facemask	Phenomenological-hermeneutical qualitative study	Narrative interviews; phenomenological-hermeneutical analysis	Everyday life with HMV, including independence, dependence, personal assistance, social encounters, and vulnerability
Khankeh et al. 2022 (31)	Iran	14 healthcare workers, 12 family members, and 2 improved patients involved in home care for ventilation-dependent patients	Grounded theory study	In-depth semi-structured interviews and supplementary documents; constant comparative analysis	Home health care process, family stress, care delegation, caregiver training, and limited professional support
Klingshirn et al. 2022 (32)	Germany	13 ventilated individuals and 18 family caregivers in the qualitative component	Convergent parallel mixed-methods study	Semi-structured interviews; framework analysis and mixed-methods integration	Care quality in private homes and shared living communities, including continuity, shared decision-making, family support, and emotional care
Mansell et al. 2020 (33)	UK	15 patients, 5 carers, and 12 healthcare professionals involved in home NIV management	Qualitative study	Semi-structured interviews and focus groups; modified framework analysis	Experiences of modem technology and remote monitoring in home NIV management
Perry et al. 2023 (34)	New Zealand	11 people with neuromuscular disorders using NIV for more than 12 months; 3 caregivers also contributed	Qualitative study guided by critical realism and contextualism	Semi-structured interviews; reflexive thematic analysis	Healthcare experiences affecting NIV access and use, including informed consent, equipment setup, mask fitting, maintenance, and patient–clinician relationships
Thorborg et al. 2023 (35)	Denmark	7 patients with ALS receiving non-invasive HMV and facing decisions about invasive HMV	Phenomenological-hermeneutical qualitative study	Semi-structured interviews; phenomenological-hermeneutical analysis	ALS patients’ experiences and needs in decision-making about invasive HMV
Wasilewski et al. 2022 (23)	Canada	8 family caregivers of ventilator-assisted individuals living at home and 5 peer mentors	Qualitative descriptive study	Weekly online peer-to-peer group chat transcripts; thematic and framework analysis	Family caregivers’ experiences of complexity, hypervigilance, role overload, and peer support
Wilson et al. 2024 (36)	UK	16 people living with MND using HMV, 10 family members, and 36 bereaved family members	Qualitative interview study using an interpretive constructivist methodology	Flexible semi-structured interviews; thematic analysis with constant comparison	End-of-life decision-making about HMV use in MND, including selective, timely, defaulted, and proactive decisions
Winther et al. 2020 (37)	Denmark	11 close relatives of people with ALS living at home with HMV or invasive HMV and formal caregivers	Phenomenological-hermeneutical qualitative study	Semi-structured individual interviews and one focus group interview; phenomenological-hermeneutical analysis	Relatives’ everyday experiences of living with ALS-related HMV, including responsibility, vulnerability, formal caregiving, and loss of private space
Yacob Hussain et al. 2025 (38)	Singapore	20 people with chronic hypercapnic respiratory failure using home NIV for at least 6 months	Descriptive qualitative study guided by Roy’s adaptation model	Face-to-face interviews; thematic analysis	Adaptation to long-term home NIV, including forced acceptance, symptom relief, side effects, mask maintenance, home integration, and travel readjustment

### Methodological quality appraisal results

According to the JBI Critical Appraisal Checklist for Qualitative Research, the overall methodological quality of the included studies was relatively good, and no Level C studies were identified. Among the 13 studies, four studies (15, 29, 34, 38) were rated as Level A, and nine studies (11, 23, 30–33, 35–37) were rated as Level B. Most studies provided adequate reporting on the congruity between the research question or study aim and the methodology, data collection methods, data analysis and interpretation of findings, representation of participants’ voices, ethical approval, and the congruity between conclusions and data interpretation. Methodological limitations mainly related to researcher positioning and reflexivity reporting. One study (36) provided unclear reporting regarding the congruity between the philosophical perspective and the research methodology. Ten studies (11, 15, 23, 30, 32, 33, 35–38) did not adequately describe the positioning of the researchers within the cultural or theoretical context. Eight studies (11, 23, 30, 32, 33, 35–37) did not describe the influence of the researchers on the research process or the interaction between the researchers and the research process, and one additional study (31) provided unclear reporting on this item. The methodological quality appraisal results are presented in Table 4.

**Table 4:** Results of the critical appraisal of the studies included.

Study	Q1	Q2	Q3	Q4	Q5	Q6	Q7	Q8	Q9	Q10	Level
Choyce et al. (29)	Y	Y	Y	Y	Y	Y	Y	Y	Y	Y	A
Esmaeili et al. (15)	Y	Y	Y	Y	Y	N	Y	Y	Y	Y	A
Ewers et al. (11)	Y	Y	Y	Y	Y	N	N	Y	Y	Y	B
Israelsson-Skogsberg et al. (30)	Y	Y	Y	Y	Y	N	N	Y	Y	Y	B
Khankeh et al. (31)	Y	Y	Y	Y	Y	Y	U	Y	Y	Y	B
Klingshirn et al. (32)	Y	Y	Y	Y	Y	N	N	Y	Y	Y	B
Mansell et al. (33)	Y	Y	Y	Y	Y	N	N	Y	Y	Y	B
Perry et al. (34)	Y	Y	Y	Y	Y	Y	Y	Y	Y	Y	A
Thorborg et al. (35)	Y	Y	Y	Y	Y	N	N	Y	Y	Y	B
Wasilewski et al. (23)	Y	Y	Y	Y	Y	N	N	Y	Y	Y	B
Wilson et al. (36)	U	Y	Y	Y	Y	N	N	Y	Y	Y	B
Winther et al. (37)	Y	Y	Y	Y	Y	N	N	Y	Y	Y	B
Yacob Hussain et al. (38)	Y	Y	Y	Y	Y	N	Y	Y	Y	Y	A

Y, yes; N, no; U, unclear; NP, not applicable.

Q1. Is there congruity between the stated philosophical perspective and the research methodology?

Q2. Is there congruity between the research methodology and the research question or objectives?

Q3. Is there congruity between the research methodology and the methods used to collect data?

Q4. Is there congruity between the research methodology and the representation and analysis of data?

Q5. Is there congruity between the research methodology and the interpretation of results?

Q6. Is there a statement locating the researcher culturally or theoretically?

Q7. Is the influence of the researcher on the research, and vice-versa,addressed?

Q8. Are participants, and their voices, adequately represented?

Q9. Is the research ethical according to current criteria and, for recent studies, is there evidence of ethical approval by an appropriate body?

Q10. Do the conclusions drawn in the research report flow from the analysis, or interpretation, of the data?

### Themes

Through repeated reading, comparison, and synthesis, five synthesized themes and 17 subthemes were developed (Table 5): (1) passive entry, repeated weighing, and active participation in HMV decision making; (2) adapting to the integration of ventilation technology into everyday family life; (3) ongoing tensions among life support, quality of life, and autonomy; (4) expansion of family caregiving responsibilities and reconstruction of the boundaries of professional care; and (5) gaps in support systems and the need for continuous support for the whole family. The correspondence between each theme and subtheme and the included studies is presented in Appendix 3.

**Table 5:** Synthesized themes and subthemes.

Synthesized themes	Subthemes
1. Passive entry, repeated weighing, and active participation in HMV decision making	a. Passive acceptance and insufficient information at the initial stage
	b. Uncertainty and repeated weighing during disease progression
	c. From reliance on professional judgment to active participation in care decisions
	d. Choices among ventilation modality, disease progression, and family caregiving capacity
2. Adapting to the integration of ventilation technology into everyday family life	a. Physical discomfort, operational difficulties, and emotional fluctuations during initial adjustment
	b. Coexistence of symptom improvement and device related adverse effects
	c. Rearrangement of the home environment and daily rhythms
	d. Balancing a sense of security, remote monitoring, and privacy ethics
3. Ongoing tensions among life support, quality of life, and autonomy	a. Maintaining an autonomous life under technological dependence
	b. Stigma, misunderstanding, and defensiveness in social participation
	c. Coexistence of improved quality of life and restricted living
	d. Sense of control at the end of life, family responsibility, and decisions about ventilation withdrawal
4. Expansion of family caregiving responsibilities and reconstruction of the boundaries of professional care	a. Transformation of family members into informal caregivers with technical responsibilities
	b. Formal caregivers as both a source of relief and pressure within the home
	c. Families assuming the role of system coordinators
	d. Care setting and professional support conditions shaping family burden
5. Gaps in support systems and the need for continuous support for the whole family	a. Insufficient discharge preparation and continuing education
	b. Insufficient psychological support and peer support
	c. Financial burden and insufficient insurance coverage
	d. Difficulties in service accessibility, equipment supply, and system navigation
	e. Shifting the target of support from the individual patient to the whole family

During the synthesis process, this study also examined the commonalities and differences between patient and caregiver perspectives, and analyzed disease type, ventilation modality, level of ventilatory dependence, and caregiving context as important contextual factors influencing experiential differences. Overall, patients with ALS/MND, and other progressive neuromuscular diseases placed greater emphasis on disease progression, future choices regarding respiratory support, invasive ventilation, and pressures related to decisions at the end of life. Patients with COPD, OHS, and chronic hypercapnia more often emphasized adaptation to long-term NIV, mask tolerance, sleep improvement, and integration of ventilation into daily life. Families involving IHMV or high ventilatory dependence more prominently reflected safety risks, heightened caregiver vigilance, and insufficient professional support. Insurance coverage, equipment supply, home nursing services, and levels of community support across different countries and healthcare systems also shaped patients’ and caregivers’ perceived burden and support needs related to HMV.

The ConQual assessment showed that the methodological dependability of all five synthesized themes was high, the credibility of the findings was moderate, and the final level of confidence was moderate for all themes. The main reason for downgrading credibility was that some synthesized themes contained both unequivocal and credible findings, rather than being composed entirely of unequivocal evidence. In addition, some included studies provided insufficient reporting on researcher reflexivity and the interaction between researchers and the research process. The ConQual assessment results for the five synthesized themes are presented in Table 6, and the full ConQual assessment results for each synthesized theme and subtheme are provided in Appendix 4.

**Table 6:** ConQual assessment of the synthesized themes.

Synthesized themes	Contributing studies (n)	Dependability	Credibility	ConQual rating
1. Passive entry, repeated weighing, and active participation in HMV decision making	8 (11, 29, 31, 34–38)	High	Moderate	Moderate
2. Adapting to the integration of ventilation technology into everyday family life	10 (11, 15, 23, 29–31, 33, 34, 37, 38)	High	Moderate	Moderate
3. Ongoing tensions among life support, quality of life, and autonomy	9 (15, 23, 29, 30, 32, 35–38)	High	Moderate	Moderate
4. Expansion of family caregiving responsibilities and reconstruction of the boundaries of professional care	8 (11, 15, 23, 31–34, 37)	High	Moderate	Moderate
5. Gaps in support systems and the need for continuous support for the whole family	13 (11, 15, 23, 29–38)	High	Moderate	Moderate

To further present the interconnection and differences between patient and caregiver experiences during home mechanical ventilation, this study summarized their main experiences and support needs within each synthesized theme. Overall, patient experiences were more focused on treatment decision-making, physical adaptation, dependence on equipment, maintenance of autonomy, social participation, and choices at the end of life. Caregiver experiences were more reflected in responsibilities for technical care, nocturnal vigilance, reorganization of family life, coordination with service systems, financial pressure, and the need for continuous support. Patient and caregiver experiences were not independent of each other; rather, they interacted within the family caregiving context and jointly shaped the long-term adaptation process associated with HMV. The correspondence between patient and caregiver experiences across the synthesized themes is presented in Table 7.

**Table 7:** Comparison of patient and caregiver experiences and support needs.

Synthesized themes	Main patient experiences	Main caregiver experiences	Representative studies
Passive entry, repeated weighing, and active participation in HMV decision making.	Insufficient information at the initial stage; uncertainty regarding ventilatory dependence, invasive ventilation, ventilation withdrawal, or care at the end of life; reevaluation of choices as the disease changes.	Participation in family discussions; assistance with or substitution in decision making; increased responsibility when patients’ capacity declines.	Perry et al. (34); Thorborg et al. (35); Wilson et al. (36).
Adapting to the integration of ventilation technology into everyday family life.	Coexistence of mask discomfort, air leakage, sleep improvement, symptom relief, and adverse effects; gradual incorporation of equipment into daily life.	Adjustment of sleep, space, and family arrangements; assistance with equipment maintenance, alarm management, and supply replenishment.	Choyce et al. (29); Ewers et al. (11); Mansell et al. (33); Yacob Hussain et al. (38).
Ongoing tensions among life support, quality of life, and autonomy.	Maintaining learning, work, travel, and autonomous living while depending on equipment and support from others; experiencing stigma and misunderstanding.	Supporting patients in maintaining daily life and social participation while bearing time, space, and emotional burdens.	Choyce et al. (29); Israelsson-Skogsberg et al. (30); Wilson et al. (36); Yacob Hussain et al. (38).
Expansion of family caregiving responsibilities and reconstruction of the boundaries of professional care.	Dependence on personal assistants, family members, or formal caregivers to maintain daily life; expectation that preferences and autonomy will be respected.	Transformation from family members into informal caregivers with technical responsibilities; coordination among hospitals, communities, equipment suppliers, and formal caregivers.	Esmaeili et al. (15); Khankeh et al. (31); Klingshirn et al. (32); Wasilewski et al. (23); Winther et al. (37).
Gaps in support systems and the need for continuous support for the whole family.	Need for continuing guidance, device adaptation, follow up, psychological support, and assured service accessibility.	Need for training, respite, financial support, psychological support, peer support, and system navigation support.	Esmaeili et al. (15); Ewers et al. (11); Khankeh et al. (31); Perry et al. (34); Wasilewski et al. (23).

#### Theme 1: passive entry, repeated weighing, and active participation in HMV decision making

##### Subtheme 1: passive acceptance and insufficient information at the initial stage

The included studies showed that patients and caregivers often began HMV during periods of disease deterioration, acute exacerbation, discharge transition, or the shock of diagnosis. At this stage, ventilation was not always initiated on the basis of full understanding and shared decision-making; rather, patients and families often entered HMV passively under the pressure of illness and clinical arrangements. For patients, HMV was not merely a therapeutic technology but also symbolized changes in disease status, bodily dependence, and adjustment of future life patterns. Insufficient information further weakened their sense of control over treatment choices.

Patient: “There was no deciding… we just had to accept it” (34).

Patient: “But how was I supposed to be able to take a decision about that at that time? It was just after I received the diagnosis?” (35).

##### Subtheme 2: uncertainty and repeated weighing during disease progression

As the disease progressed and the need for ventilatory support increased, patients’ and families’ attitudes toward HMV often changed dynamically. In progressive diseases such as ALS/MND in particular, patients had to repeatedly weigh life prolongation, quality of life, family caregiving capacity, choices regarding invasive ventilation, and end-of-life care arrangements. Patients’ judgments about what living conditions remained acceptable were not fixed but continued to change with disease stage, physical adaptation, and family circumstances.

Patient: “Yes, the decision so far is… not IHMV. However, I always hurry to say ‘so far’…” (35).

Patient: “Now, five years ago, if you’d asked me how I would feel about the position I’m in now, I’d probably say, oh God, I could not live with that. But I can live with that and I can enjoy life with that” (36).

##### Subtheme 3: from reliance on professional judgment to active participation in care decisions

As treatment continued, some patients gradually moved from passive acceptance to active participation, beginning to express preferences regarding ventilation modality, duration of use, place of care, and treatment boundaries. Some caregivers also became increasingly familiar with equipment maintenance, airway management, and emergency response during long-term caregiving, thereby taking on a greater supportive role in treatment adjustment and care arrangements. In this way, family-based HMV care was no longer merely the implementation of clinical arrangements but gradually became a process in which patients, caregivers, and professionals jointly adjusted and managed treatment.

Caregiver: “I do all the work now. Even better than the nurses. The evidence is that the patient has no infection, no bedsore, nothing” (31).

Patient: “All of a sudden I’m now on NIV for 20 + hours per day, and I’m thinking about asking for ventilation to be withdrawn sometime during this summer” (36).

##### Subtheme 4: choices among ventilation modality, disease progression, and family caregiving capacity

HMV decision-making involved not only whether to use a ventilation device but also specific choices regarding non-invasive or invasive ventilation, device adaptation, adjustment of usage duration, and whether home care should continue. Different underlying diseases, ventilation modalities, and levels of ventilatory dependence shaped the decision pressures faced by patients and caregivers. Patients with neuromuscular diseases may gradually require longer hours of ventilatory support as the disease progresses. Patients with ALS/MND were more likely to face decisions related to tracheostomy, withdrawal of ventilation, or end-of-life care, whereas patients receiving long-term NIV were more concerned with device adaptation, comfort, and integration into daily life.

Patient: “we tried one equipment, and if any problem occurred, if we were having difficulties, we talked to the doctor and the doctor talked to the homecare provider” (29).

Patient: “It wasn’t discussed whether that was the only option, they said ‘we have got this for you’” (34).

#### Theme 2: adapting to the integration of ventilation technology into everyday family life

##### Subtheme 1: physical discomfort, operational difficulties, and emotional fluctuations during initial adjustment

When HMV was transferred into the home, patients and caregivers had to transform ventilation treatment that had previously been led by professionals into daily care practice within the family setting. The initial stage was often accompanied by mask discomfort, operational difficulties, sleep disruption, the need to respond to alarms, and psychological resistance to dependence on equipment. For patients, receiving long-term ventilation was not only a matter of learning how to operate the device but also involved gradual adaptation to physical discomfort, treatment dependence, and changes in lifestyle. This process was therefore often accompanied by emotional changes, including a sense of loss, resistance, and gradual adjustment.

Patient: “At first, the nurses took over everything. And then, all of a sudden, they told me, ‘Try doing it yourself.’ And yeah, that did not work for me at first. Not at all. I was, like, blocked inside” (11).

Patient: “I was disappointed because I (must) use all these things (mask and ventilator) for my whole life. You know, it will be quite a long time. Yes, but then, because of my sickness, I have no choice” (38).

##### Subtheme 2: coexistence of symptom improvement and device related adverse effects

Several studies showed that HMV could alleviate dyspnea, reduce headaches and fatigue, improve sleep quality and daytime functioning, and help patients feel that their physical condition and quality of life had improved. However, long-term use could also lead to mask pressure, skin injury, pain, air leakage, noise, and an increased burden of maintenance. Patients’ experiences of HMV therefore reflected a clear tension: they relied on HMV for symptom relief while continuing to endure equipment-related discomfort.

Patient: “I was getting a rash from the mask… I was at my wits end with the rash” (29).

Patient: “When I wake up, I do not have a headache anymore, and I do not have that very frustrated feeling when I wake up” (38).

##### Subtheme 3: rearrangement of the home environment and daily rhythms

HMV not only changed patients’ mode of treatment but also reshaped domestic space, sleep arrangements, toileting routines, travel plans, and the rhythm of everyday life. Patients had to adjust daily details around device placement, tubing connections, and periods of use. Caregivers were also drawn into an ongoing process of adaptation through nocturnal care, equipment management, and changes in sleeping arrangements. In this way, the home was transformed from an ordinary living space into a setting that combined both everyday life and care functions.

Patient: “I put it on the rack-like trolley and then bring the whole thing to the toilet door. Then I go and do (urinate)” (38).

Caregiver: “It’s quite impossible to sleep in the same room, so my mom sleeps in a separate room. Yeah, and I’ll be at the hall for the night” (38).

##### Subtheme 4: balancing a sense of security, remote monitoring, and privacy ethics

Remote monitoring, professional follow-up, and feedback from device data could strengthen patients’ and caregivers’ sense of security by helping them believe that changes in the patient’s condition could be detected promptly and addressed by professionals. At the same time, remote monitoring could also alter the boundaries of observation, response, and responsibility in family care. Patients and caregivers were therefore concerned not only about whether device data could be uploaded but also about whether clear, timely, and predictable professional support was available in response to those data. When the original studies reported privacy or ethical concerns, these findings suggested that technological support, while enhancing a sense of security, also needed to respect privacy boundaries and informed awareness within the family context.

Caregiver: “If there was a problem that I could not pick up, it would certainly pick up on the modem, and they would phone us and say it needs altering or whatever. That makes me feel far more confident” (33).

#### Theme 3: ongoing tensions among life support, quality of life, and autonomy

##### Subtheme 1: maintaining an autonomous life under technological dependence

HMV meant that patients remained dependent on equipment, family members, personal assistants, or professional caregivers, while also making it possible for them to continue education, work, family life, and social participation. The core tension in patients’ experiences was not a simple contrast between independence and dependence but the effort to maintain a sense of control over daily arrangements, bodily care, and personal choices under conditions of substantial dependence on technology and support from other people.

Patient: “My PCAs means a lot in my daily life. They give me all opportunities to live a life the way I want, to be in charge, and control all aspects of life instead of someone else is doing it for me” (30).

##### Subtheme 2: stigma, misunderstanding, and defensiveness in social participation

In public spaces, patients were often misunderstood, ignored, or infantilized because of their use of wheelchairs, ventilation equipment, or personal assistants. Such experiences affected their social participation, self-esteem, and identity. Young HMV users in particular emphasized that physical dependence did not imply a lack of cognitive, communicative, or decision-making capacity. They wished to be treated as individuals with agency rather than being defined by their equipment or care needs.

Patient: “It’s very often that people who see a wheelchair make their own decision that he’s not able to talk or think, they talk over my head turning directly to my PCAs” (30).

##### Subtheme 3: coexistence of improved quality of life and restricted living

HMV could improve respiratory symptoms, sleep, energy levels, and life expectancy, but it could also cause physical discomfort, affect intimate relationships, alter personal image, and restrict family activities. Patients’ evaluations of quality of life therefore did not simply reflect improvement or decline but represented a complex experience shaped by symptom relief, functional maintenance, and equipment-related restrictions.

Patient: “…it’s a massive cog, because it has had a good effect on my life…it’s been positive without a shadow of a doubt” (29).

Patient: “I do not think it’s helped our sex life because he will not sleep in the same room as me now” (29).

##### Subtheme 4: sense of control at the end of life, family responsibility, and decisions about ventilation withdrawal

In progressive diseases such as ALS/MND, HMV was also related to end-of-life care, advance care planning, and decisions about ventilation withdrawal. Some patients were reluctant to define future treatment boundaries too early because their judgments about what would constitute an acceptable living condition might change as the disease progressed. Others maintained a sense of control at the end of life by actively discussing ventilation withdrawal, preferred place of death, or treatment boundaries. This experience was mainly observed among patients with ALS/MND or a high level of ventilatory dependence and should not be directly generalized to all patients using nocturnal NIV or those with low levels of dependence.

Patient: “I do not want to make decisions like that now, because when the time comes I might feel quite different about it” (36).

Patient: “All of a sudden I’m now on NIV for 20 + hours per day, and I’m thinking about asking for ventilation to be withdrawn sometime during this summer” (36).

#### Theme 4: expansion of family caregiving responsibilities and reconstruction of the boundaries of professional care

##### Subtheme 1: transformation of family members into informal caregivers with technical responsibilities

The responsibilities undertaken by family caregivers in HMV care far exceeded general assistance with daily living and often involved airway management, equipment maintenance, alarm management, nocturnal observation, service coordination, and risk assessment. As caregiving continued, family members gradually moved beyond their role as relatives and became informal caregivers undertaking complex technical tasks. This transformation could enhance the family’s capacity to respond to care demands, but it could also alter family relationships and increase caregivers’ physical burden, psychological strain, and sense of responsibility.

Caregiver: “I do all the work now. Even better than the nurses. The evidence is that the patient has no infection, no bedsore, nothing” (31).

Caregiver: “Well, I made it clear that I would not be part of it (personal care), but I got involved” (37).

##### Subtheme 2: formal caregivers as both a source of relief and pressure within the home

Formal caregivers, home nurses, or personal assistants could take on technically demanding care tasks and enhance the family’s sense of security. However, their long-term presence in the home could also alter private space, intimate relationships, and family boundaries. In situations requiring continuous care, domestic living space could be reshaped by ongoing caregiving activities. Family members relied on formal caregivers for respite and a sense of security, but they could also feel that their privacy was continuously intruded upon and that family life was being reorganized.

Caregiver: “It is a great invasion – that suddenly there are people in your home all the time” (37).

Caregiver: “All the time, you have to remember that it is your home and that you are the one who decides how things are going to be. Not the other way around” (37).

##### Subtheme 3: families assuming the role of system coordinators

When coordination among hospitals, community services, equipment suppliers, and home nursing services was insufficient, caregivers were often forced to become system coordinators. They not only had to care for the patient but also contact institutions, coordinate personnel, follow up on equipment maintenance, address service gaps, and assess risks. Such coordination work increased caregivers’ time burden and psychological stress and also indicated that families had assumed some continuity-of-care functions that should have been provided by the HMV care system.

Caregiver: “somehow it is expected that we are the coordinators between everything and everyone” (37).

##### Subtheme 4: care setting and professional support conditions shaping family burden

Experiences of HMV care were jointly influenced by care setting, intensity of professional support, organization of services, and resource accessibility. Staffing, service coordination, and the degree of family involvement varied across care settings, but family burden was not determined simply by care setting. The included studies showed that continuity of professional support, clear boundaries of responsibility, service accessibility, and respect for family preferences and role allocation all influenced family caregiving burden and the sense of security.

Caregiver: “It is an enormous amount of work – all the places you have to call all the time to follow up on things and all the times we need help, and nobody can help us” (37).

#### Theme 5: gaps in support systems and the need for continuous support for the whole family

##### Subtheme 1: insufficient discharge preparation and continuing education

Patients and caregivers usually required systematic training in device operation, alarm management, tubing cleaning, mask maintenance, emergency response, and recognition of long-term risks. However, the included studies showed that the education received by families was often concentrated on short-term guidance at treatment initiation or around discharge and was insufficient to address the complex problems encountered in real-world home settings. In families caring for patients receiving IHMV or with high levels of ventilatory dependence in particular, caregivers’ ability to respond to emergencies and access to continuing education were especially critical, yet such support could still be inadequate.

Caregiver: “The whole technical aspect… that’s obvious, depending on how technically skilled you are. But the adjustment [you have to make] to living with something like that… that probably needs a bit more support from someone, yeah” (11).

Caregiver: “I only know about the issue to the extent that I have heard about it. I have not done or seen it done yet. Now I need to go and ask. Now that you have raised this, I’ve come to think of it. If it happens, what then?” (15).

##### Subtheme 2: insufficient psychological support and peer support

Long-term HMV care was not only a technical task but also a sustained psychological and emotional burden. Patients could experience fear and a sense of loss related to dependence on equipment, uncertainty about the future, and bodily changes. Caregivers could feel exhausted when the patient’s condition did not improve, daily life was disrupted, opportunities for respite were lacking, and long-term responsibility persisted. Peer support could provide caregivers with validation of their experiences and emotional buffering, but in the care contexts presented in the existing studies, such support still did not fully meet families’ continuing needs.

Caregiver: “My life’s routine has become messed up completely. It’s been months since I’ve taken a trip or had any fun. Because my patient’s consciousness has not changed, not changed at all, it’s remained the same, I feel that all my efforts have been useless. I’ve gotten really tired” (15).

Caregiver/Mentor: “Support from anyone can bring instant relief!” (23).

##### Subtheme 3: financial burden and insufficient insurance coverage

Financial pressure was an important factor affecting the sustainability of HMV family care. Equipment rental, home nursing services, medical services, consumables, transportation, and loss of income could all increase family burden. In some healthcare systems, insufficient insurance coverage and service payment pressures created tension for families between maintaining care quality and bearing financial costs. Financial burden not only affected families’ quality of life but could also limit their ability to obtain professional care and continuous support.

Caregiver: “The daily wage of his home nurse are 40 dollars, the cost of renting a ventilator is 67 dollars, and the other equipment are also very expensive. All our monthly salary is spent entirely on equipment and paying for home nurses” (15).

Caregiver: “I am under a lot of pressure. The one who spends, expects improvement. Our patient has no sign of recovery. The steady-state is painful for the patient’s family” (31).

##### Subtheme 4: difficulties in service accessibility, equipment supply, and system navigation

Across different healthcare systems, patients and caregivers could face problems related to equipment maintenance, mask replacement, access to professional advice, coordination across institutions, and inconsistent service standards. When service responses were delayed or professionals could not address complex problems in home ventilation in a timely manner, families were likely to experience a reduced sense of security and bear additional pressure during device replacement, equipment malfunction management, and emergencies. These experiences indicated that families receiving HMV needed not only equipment itself but also stable, accessible, and continuous professional support.

Caregiver: “My husband needed the respirator to live. It’s a bit scary when help is 2 h away” (23).

Caregiver: “I have had lots of pressure to change ventilators because they no longer want to supply/maintain the ventilator he is on now” (23).

##### Subtheme 5: shifting the target of support from the individual patient to the whole family

The included studies showed that support needs related to HMV were not limited to the individual patient or device operation, but extended to the entire family caregiving system. Patient-related experiences mainly pointed to the need for continued device adaptation, follow-up feedback, and support for integration into daily life. Caregiver-related experiences focused on skills training, respite support, financial assistance, peer communication, and system navigation. The focus of family support needs varied across disease types and levels of ventilatory dependence. Families facing progressive neuromuscular diseases and high levels of ventilatory dependence placed greater emphasis on safety management, end-of-life decision-making, and assessment of caregiving capacity, whereas long-term NIV users were more concerned with adherence, comfort, and integration into daily life. These findings suggest that HMV support needs extended beyond management of the individual patient and involved continuous support for the family as a unit.

Caregiver: “At most I can sit in the hall outside his room but I need to see him to make sure he does not have trouble breathing from a mucus plug. I would take the time to be with friends if I could” (23).

## Discussion

This study systematically synthesized the experiences and support needs of patients receiving HMV and their caregivers during home treatment. The findings showed that HMV is not merely a respiratory support technology but a long-term care process embedded in disease progression, family life, caregiving relationships, and healthcare service systems. The experiences of patients and caregivers extended across multiple dimensions, including treatment decision-making, technological adaptation, quality of life, maintenance of autonomy, expansion of family caregiving responsibilities, and the need for continuous support. These findings suggest that challenges related to HMV are not limited to device use or treatment adherence but are jointly shaped by illness trajectories, levels of ventilatory dependence, family caregiving capacity, availability of professional support, and allocation of institutional resources.

Previous studies on the experiences of patients receiving HMV have shown that, while HMV can improve symptoms, support home living, and help maintain a certain level of quality of life, it may also involve passive acceptance, uncertainty, difficulties in technological adaptation, care coordination pressures, and ongoing information needs (11, 29, 30, 34–38). Compared with previous research (39), the findings of this study are generally consistent but further extend existing understanding by integrating the perspectives of both patients and caregivers and by emphasizing the influence of disease background, ventilation modality, level of ventilatory dependence, family caregiving capacity, and healthcare system resources on experiential differences. Therefore, this study not only presents the individual experiences of patients receiving HMV but also further reveals the role of the family caregiving system and continuous support pathways in the long-term management of HMV.

From a theoretical perspective, the findings of this study can be understood through an integrated lens of chronic illness trajectory theory and the social ecological model (40–42). Chronic illness trajectory theory emphasizes that chronic disease management is not a static process but is continuously adjusted according to disease stage, functional status, treatment goals, and care needs (40, 41). The experiences of patients receiving HMV and their families reflected this dynamic feature. Some patients started HMV during disease deterioration, acute exacerbation, or discharge transition, and the early stage was often accompanied by insufficient information and passive acceptance. As the disease progressed, patients and families needed to continuously weigh symptom relief, ventilatory dependence, quality of life, caregiving capacity, and future treatment choices. When the disease entered a stage of high dependence or end-of-life care, related decisions further involved invasive ventilation, ventilation withdrawal, advance care planning, and proxy decision-making (30, 34–36).

The social ecological model further suggests that HMV experiences are not determined solely by individual disease status but are jointly influenced by multiple levels of factors, including the individual, family, service, and institutional levels (42). Patients’ disease type, physical function, and psychological status; family-level caregiving resources and financial capacity; service-level professional teams, equipment supply, and follow-up support; and institutional-level insurance coverage, community services, and healthcare system structures may all influence the adaptation process and sustainability of HMV care (24). Therefore, HMV support models should not focus only on individual patients or equipment management but should be understood as a continuous care process that extends across the illness trajectory, family system, and healthcare service system.

This study further suggests a possible pathway underlying HMV experiences. Disease progression, acute exacerbation, or discharge transition often represents an important context in which patients enter HMV treatment (18). When sufficient explanation and shared decision-making are lacking during treatment initiation, patients and caregivers may develop uncertainty and a sense of passive acceptance (34). After treatment is transferred into the home, device operation, alarm management, mask discomfort, sleep changes, spatial adjustment, and consumable management gradually reshape the structure of everyday family life, reorganizing domestic life around medical care activities to some extent (11). When continuing training, professional follow-up, and equipment support are insufficient, some responsibilities that should have been undertaken by professional systems may be further transferred to the family. In this process, caregivers gradually shift from family members to informal caregivers undertaking complex technical tasks and assume responsibilities for risk monitoring, system coordination, and emergency response (43). While long-term technological dependence may improve symptoms and prolong life, it may also increase restrictions on autonomy, social isolation, family tension, and psychological pressure. Taken together, HMV experiences may form a continuous process from disease progression or treatment initiation to family adaptation, transfer of caregiving responsibilities, and expansion of support needs. The synthesized themes in this study are therefore not independent categories of experience but collectively constitute a dynamic chain of long-term HMV care.

The experiences of patients and caregivers differed across disease backgrounds. Patients with ALS/MND and other progressive neuromuscular diseases placed greater emphasis on irreversible disease progression, choices regarding respiratory support, declining communication ability, and pressures related to end-of-life decision-making (12). For these patients, HMV was not only meaningful for symptom relief but was also closely related to life prolongation, functional loss, family proxy decision-making, and choices about ventilation withdrawal (36). Therefore, their support needs placed greater emphasis on early communication, staged shared decision-making, advance care planning, and integration of palliative care. By contrast, the experiences of patients with chronic obstructive pulmonary disease, obesity hypoventilation syndrome, chronic hypercapnia, and other conditions requiring long-term NIV were more focused on adaptation to long-term NIV, mask tolerance, sleep improvement, symptom control, and integration into daily life (38, 44). These patients may not continuously face rapid end-of-life decisions, but they may be more likely to experience insufficient comfort, fluctuations in adherence, and inadequate follow-up support during long-term use (45). Therefore, HMV care strategies should be stratified according to the rate of disease progression, prognostic characteristics, and ventilation goals, because a single care model may not adequately respond to the support needs of different patients and families.

Ventilation modality and level of dependence also influenced the experiences of patients and caregivers. Patients receiving non-invasive ventilation often had to cope with mask pressure, air leakage, oral or nasal dryness, sleep disturbance, and difficulties in device adaptation, although autonomy in daily activities and social participation could still be maintained under certain conditions. By contrast, patients receiving IHMV or near-continuous ventilation were more dependent on family members, formal caregivers, and professional teams, and their care involved higher risks, greater technical complexity, and greater demands for emergency management (15, 22). For caregivers, airway management, suctioning, alarm management, and continuous observation related to invasive ventilation could lead to heightened vigilance, sleep deprivation, and psychological burden (24). Duration of ventilation use may also shape experiential characteristics. New users may be more likely to experience fear, uncertainty, and unfamiliarity with device operation, whereas long-term users may have developed certain adaptation strategies but may also accumulate chronic fatigue, financial pressure, and caregiving burnout (24, 46). Because the included studies did not report HMV duration and level of dependence consistently, this study was unable to conduct a more detailed stratified synthesis. However, these differences suggest that future research should treat ventilation modality, daily duration of use, and level of dependence as core stratification variables.

From the perspective of transferability, the synthesized findings of this study should not be understood as homogeneous conclusions applicable to all HMV populations. Instead, they should be interpreted in a stratified manner according to disease type, ventilation modality, level of ventilatory dependence, and degree of caregiving dependence. Some findings may have relatively high cross-context transferability, including insufficient information and the need for shared decision-making during treatment initiation, difficulties in device adaptation during the early home period, caregiver training needs, professional follow-up needs, equipment and consumable support, psychosocial support, difficulties in service navigation, and the need for continuous support for the family as a unit (10). These findings may be broadly applicable to long-term NIV, IHMV, and HMV patients and families across different underlying disease backgrounds because once the treatment setting extends from the hospital to the home, patients and caregivers usually need to jointly face device use, symptom monitoring, allocation of caregiving responsibilities, accessibility of professional support, and reorganization of family life (13, 47). By contrast, some findings are more specific to particular illness trajectories, ventilation modalities, or high-dependence caregiving contexts and should not be directly generalized to all patients receiving HMV. Decisions about ventilation withdrawal, advance care planning, sense of control at the end of life, family proxy decision-making, and repeated weighing of invasive ventilation choices are more closely related to progressive neuromuscular diseases such as ALS/MND, IHMV, or near-continuous ventilatory dependence (36, 48). Caregiving burdens related to airway management, suctioning, infection prevention, alarm management, and continuous heightened vigilance are more prominent in families caring for patients receiving IHMV or with high levels of ventilatory dependence (15, 49). Conversely, mask tolerance, air leakage, improved sleep quality, long-term adherence, and comfort adjustment are more applicable to long-term NIV users, especially those with relatively stable or slowly progressive conditions such as COPD, obesity hypoventilation syndrome, and chronic hypercapnia (5, 50). Therefore, the clinical application of the findings of this study should avoid a uniform HMV support pathway and should instead be contextually translated according to disease type, ventilation modality, daily duration of ventilation, level of ventilatory dependence, caregiver capacity, and healthcare system resources.

Home-based and technology-supported care should also be further emphasized in HMV continuous support pathways. The findings of this study showed that remote monitoring, professional follow-up, and feedback from device data could enhance patients’ and caregivers’ sense of security to some extent. However, technology itself cannot replace continuous, responsive, and person-centered professional support. Existing qualitative studies on home-based telemedicine experiences in chronic disease care suggest that telemedicine can better promote patients’ sense of security, self-management, continuity of care, and treatment adherence only when it is supported by reliable technology, clear monitoring goals, and stable patient-professional relationships (51, 52). Therefore, remote monitoring and digital follow-up related to HMV should not be designed merely as tools for device data upload or parameter monitoring. Instead, they should be integrated into a family-centered continuous care system to support identification of symptom changes, feedback on ventilation comfort, management of equipment and consumable problems, responses to caregivers’ questions, risk warning, psychological support, and linkage to referral or home visits when necessary (53). At the same time, the design of remote monitoring and digital follow-up should remain person-centered, pay attention to the real-world experiences of patients and caregivers in the home context, and consider privacy protection, informed consent, digital health literacy, and differences in family resources, so as to avoid further transferring new technological responsibilities to patients and caregivers (52). In addition, digital follow-up and remote support should not rely solely on physiological indicators or device parameters, but should also incorporate patient narratives, caregiver feedback, and individual life goals to identify needs that may not be captured by standardized monitoring indicators (54). Future HMV intervention development could further integrate remote monitoring, digital follow-up, patient narrative feedback, and family resource navigation into continuous care pathways, thereby forming a service model that combines technological support capacity with family-centered and person-centered care.

National and healthcare system contexts further shaped the formation of HMV experiences. The included studies were conducted in the United Kingdom, Iran, Germany, Denmark, Sweden, New Zealand, Canada, and Singapore, and these countries differed in equipment supply, home nursing services, insurance payment, community follow-up, and access to professional teams. In contexts where professional support was relatively stable and follow-up pathways were clear, patients and caregivers were more likely to experience a sense of security and continuity of care (11). By contrast, when insurance coverage was insufficient, equipment supply was unstable, community services were weak, or cross-institutional collaboration was inadequate, families often had to assume greater coordination responsibilities and financial pressure. Socioeconomic status and healthcare support conditions were therefore not merely background information but important contextual factors influencing HMV experiences and their interpretation (15, 22). Families with relatively sufficient resources may be more able to adapt to equipment use and navigate services, whereas the caregiving difficulties of families with limited resources may be more complex and may remain underrepresented in existing studies (23). Therefore, interpretation of HMV experiences should not focus only on patient adherence or caregiver capacity but should also consider the influence of institutional resources, payment mechanisms, and continuity of services on family caregiving burden.

The experiences of patients and caregivers were interrelated but had different emphases. Patient experiences were more focused on treatment choices, physical adaptation, dependence on equipment, maintenance of autonomy, social participation, and sense of control at the end of life. Caregiver experiences were more reflected in responsibilities for technical care, nocturnal vigilance, reorganization of family life, system coordination, financial pressure, and the need for continuous support (29–31, 36–38). Patients usually sought to maintain autonomous living as much as possible under conditions of ventilatory dependence, whereas caregivers had to assume greater responsibility for safety monitoring and risk control (30, 31, 37). Their goals were not always fully aligned: patients may have placed greater emphasis on autonomy, quality of life, and a sense of control over everyday life, whereas caregivers may have been more concerned with safety, feasibility of care, and prevention of emergencies (30, 31, 36, 37). After formal caregivers or professionals entered the home, they could reduce some caregiving burden, but they could also alter the privacy of domestic space and role boundaries, placing the home in an intertwined state between a living space and a medical care setting (30, 37). Therefore, HMV care should not only assess patients’ symptoms and device use but also evaluate caregiver burden, family relationships, caregiving boundaries, and respite needs.

Based on the above findings, this study proposes a preliminary conceptual model for continuous support in home mechanical ventilation. This model uses the illness trajectory as the temporal axis and patients, caregivers, professional teams, and the institutional environment as support levels. It emphasizes that HMV care should cover continuous stages, including treatment initiation, discharge transition, early home adaptation, long-term follow-up, disease progression, and end-of-life care. To enhance the clinical operability of this model, this study further translated it into a staged assessment and support pathway. During treatment initiation, structured information provision and shared decision-making should be conducted to clarify ventilation goals, expected benefits, potential burdens, alternative options, and long-term caregiving responsibilities. Patient preferences, treatment goals, and caregiver preparedness should also be assessed. During discharge transition, family caregiving capacity, home space conditions, equipment configuration, consumable supply, emergency response capacity, and accessibility of community resources should be systematically assessed. Individualized discharge plans, device operation checklists, alarm management procedures, and follow-up pathways should then be developed (55). During early home adaptation, telephone follow-up, remote monitoring, home visits, or outpatient reassessment may be used to continuously evaluate ventilation comfort, symptom changes, sleep quality, device-related problems, and caregiver anxiety. Based on feedback, device interfaces, educational content, and the frequency of support should be adjusted in a timely manner (56).

During the long-term follow-up stage, caregiver burden screening, psychosocial support, peer support, referral to respite services, financial and resource navigation, and support for patient autonomy and social participation should be incorporated into routine management. During disease progression, treatment goals, ventilation modality, place of care, intensity of professional support, and sustainability of family caregiving should be dynamically reassessed according to changes in functional status, level of ventilatory dependence, symptom burden, and family caregiving capacity. Palliative care, anticipatory care planning, and family communication support should also be introduced in a timely manner (57). During the end-of-life care stage, advance care planning, discussions about ventilation withdrawal, symptom relief, support for family proxy decision-making, and bereavement support should be integrated into the continuous care pathway on the basis of respect for patients’ preferences and family contexts, rather than being initiated passively only in crisis situations.

Therefore, this conceptual model can be used not only to interpret the experiences of patients receiving HMV and their caregivers but also as a framework for future intervention development. Future studies may use this model to develop staged, family-centered continuous support programs for HMV and further evaluate their effects on patients’ sense of security, quality of life, caregiver burden, service accessibility, and sustainability of care.

The findings of this study also resonate with research on palliative care for life-limiting respiratory diseases. Previous studies have indicated that non-invasive respiratory support in palliative care can relieve dyspnea, but it may also prolong treatment decision-making at the end of life and introduce ethical complexity involving treatment goals, patient autonomy, and family responsibilities (58). This view is consistent with the experiences of patients with progressive neuromuscular diseases and their families identified in this study (12, 59). In this study, patients with ALS/MND and their families repeatedly weighed ventilatory dependence, choices regarding invasive ventilation, ventilation withdrawal, sense of control at the end of life, and family responsibilities, suggesting that HMV care should not be separated from palliative care. Palliative care should not be initiated only when advanced disease, ventilation withdrawal discussions, or end-of-life decisions arise, but should instead be embedded earlier in the HMV illness trajectory and ventilatory treatment process (60).

At the treatment initiation stage, early palliative care can help patients and caregivers understand the goals of HMV, potential benefits, treatment limitations, possible burdens, and future caregiving responsibilities through treatment goal communication and shared decision-making (61). During home adaptation and long-term follow-up, palliative care can address symptom experiences such as dyspnea, fatigue, anxiety, sleep disturbance, mask discomfort, and perceived dependence on equipment. It can also reduce long-term family caregiving pressure through caregiver burden screening, psychosocial support, family communication, linkage to respite resources, and service navigation (62). During disease progression, early palliative care can support patients, caregivers, and professional teams in continuously reassessing treatment goals and discussing worsening ventilatory dependence, transition from non-invasive to invasive ventilation, place of care, emergency management plans, and the boundaries of care that families can sustain. It can also support the gradual development of anticipatory care plans focused on anticipating symptom deterioration, responding to acute events, coordinating care resources, and arranging place of care (57). For patients with ALS/MND, IHMV, or high levels of ventilatory dependence, advance care planning discussions should also be conducted at an appropriate time based on patients’ values and treatment preferences, in order to clarify key issues such as future invasive ventilation, ventilation withdrawal, proxy decision-making, and end-of-life care arrangements.

Therefore, the integration of palliative care into HMV care should shift from an end-of-life–oriented approach to a whole-course support approach. Such integration does not mean abandoning ventilatory treatment. Rather, it means maintaining respiratory support while continuously addressing symptom control, quality of life, patient autonomy, caregiver burden, alignment of treatment goals, and family caregiving capacity (63). For different HMV populations, the focus of palliative care involvement should also be differentiated. For long-term NIV users, greater emphasis may be placed on symptom control, comfort improvement, adherence support, and enhancement of quality of life. For patients with progressive neuromuscular diseases, those receiving IHMV, or those in high-dependence caregiving contexts, treatment goal communication, anticipatory care planning, advance care planning, family proxy decision-making, and preparation for end-of-life care should be incorporated earlier.

### Implications for clinical practice

This study suggests that HMV nursing practice should shift from equipment delivery and one-time operation training toward continuous support for the family as a unit across the illness trajectory. At treatment initiation, nurses should help patients and families understand the treatment goals, applicable boundaries, potential benefits, possible burdens, and long-term caregiving responsibilities associated with HMV. They should also support staged shared decision-making according to disease trajectory, ventilation modality, and level of dependence. For patients with progressive neuromuscular diseases such as ALS/MND, invasive ventilation choices, preferences regarding ventilation withdrawal, advance care planning, and palliative care should be incorporated into ongoing communication rather than being addressed temporarily during a crisis.

During discharge transition and long-term home care, family caregiving capacity and caregiver burden should be core components of assessment. Nurses need not only to provide training in device connection, mask fitting, alarm management, consumable management, and emergency response but also to assess caregivers’ understanding, home space conditions, nocturnal care arrangements, financial pressure, psychological burden, and available community resources. For families caring for patients receiving IHMV or with high levels of ventilatory dependence, more intensive training should be provided in airway management, suctioning, infection prevention, and emergency simulation, with continuous reinforcement through follow-up, home assessment, and repeated feedback.

At the organizational and policy levels, collaborative mechanisms should be established among hospitals, communities, equipment suppliers, home nursing agencies, and insurance payers. The responsible parties for equipment maintenance, consumable supply, remote monitoring feedback, emergency response, and referral pathways should be clearly defined. Quality evaluation of HMV services should also expand beyond device use and clinical outcomes to include patient autonomy, caregiver burden, family adaptation, service accessibility, digital follow-up experiences, and quality of continuous care.

### Strengths and limitations

This study has several strengths. First, it included 13 qualitative studies and integrated multiple perspectives, including those of patients, current family caregivers, and bereaved family members, which helped provide a relatively comprehensive understanding of experiences and support needs during home treatment with HMV. Second, this study clearly defined the inclusion boundaries of HMV and considered disease type, ventilation modality, duration of use or level of dependence, caregiver type, national or healthcare system context, and information related to socioeconomic conditions and healthcare support. This improved the transparency of participant definition and the interpretation of heterogeneity. Third, this study used the JBI approach to qualitative meta-synthesis and applied ConQual to assess the quality of evidence for the synthesized themes. It also compared patient and caregiver experiences, which helped strengthen the systematic nature and traceability of evidence interpretation.

This study also has several limitations. First, although 13 studies were included, the available qualitative evidence remains limited in relation to the high heterogeneity of HMV populations. On the one hand, this is related to the strict conceptual definition of HMV used in this study and the exclusion of studies focusing solely on oxygen therapy, acute mechanical ventilation in hospital settings, or CPAP for uncomplicated OSA. On the other hand, it also reflects the insufficient amount of primary qualitative research on the experiences of patients receiving home mechanical ventilation and their caregivers. Second, the included studies were mainly conducted in Europe, North America, and some Asian countries, with limited evidence from low-income and lower-middle-income regions. Therefore, the transferability of the findings across different cultures, payment systems, and family caregiving traditions should be interpreted with caution. Third, the included studies involved different disease contexts, ventilation modalities, and levels of dependence. Although this study provided a structured discussion of these differences, the thematic synthesis may still have weakened some subtle differences related to specific diseases or ventilation modalities. Fourth, the original studies did not report HMV duration, daily ventilation time, intensity of caregiving support, socioeconomic status, healthcare support conditions, and researcher reflexivity in a fully consistent manner, which may have affected the transparency and robustness of the synthesized evidence. Finally, this study focused on the experiences and support needs of patients and caregivers rather than the clinical effectiveness of HMV. Therefore, the findings should not be directly generalized to outcomes such as survival, readmission, or objective improvement in quality of life.

Future research should further conduct qualitative studies stratified by disease type, ventilation modality, level of dependence, and healthcare system. In particular, more comparative research is needed among families affected by progressive neuromuscular diseases, long-term NIV related to chronic respiratory failure, invasive ventilation, and high ventilatory dependence. In terms of study design, longitudinal qualitative research could be used to follow changes in the experiences of patients and caregivers from treatment initiation, discharge transition, and home adaptation to disease progression or the end-of-life stage, thereby addressing the limitation that cross-sectional interviews may not adequately capture dynamic trajectories. Future studies should also report patients’ socioeconomic background, healthcare support conditions, duration of HMV use, and daily level of ventilatory dependence more systematically to improve the interpretability and transferability of evidence. In intervention research, the preliminary conceptual model of continuous support proposed in this study may be used to develop and evaluate the feasibility and effects of family-centered continuous support interventions involving shared decision-making, family caregiver training, remote monitoring and digital follow-up, peer support, respite services, resource navigation, and integration of palliative care.

## Conclusion

This study systematically synthesized the experiences and support needs of patients receiving HMV and their caregivers during home treatment. The findings showed that HMV is not only a long-term respiratory support technology but also a continuous care process deeply embedded in disease progression, family life, and healthcare service systems. Patients and caregivers faced multiple challenges related to treatment decision-making, technological adaptation, maintenance of autonomy, expansion of family caregiving responsibilities, and insufficient support systems. Disease type, ventilation modality, level of ventilatory dependence, socioeconomic conditions, and healthcare system context all influenced HMV experiences and related support needs. Therefore, HMV care should shift from individual patient management and guidance on device use toward a model of continuous support for the family as a unit. In clinical practice, shared decision-making, caregiver training, professional follow-up, psychological support, resource navigation, and integration of palliative care should be continuously provided across treatment initiation, discharge transition, home adaptation, long-term follow-up, disease progression, and the end-of-life stage. This shift may help improve the sense of security, quality of life, and sustainability of care for both patients and caregivers and provide a basis for developing continuous care pathways for HMV.
