# Supplementary material for: Experiences and support needs of patients receiving home mechanical ventilation and their caregivers: a qualitative meta-synthesis
Source: Front Public Health. 2026 Jul 2;14:1793552. doi: 10.3389/fpubh.2026.1793552 (PMC13373040; doi:10.3389/fpubh.2026.1793552)
Supplement: Supplementary file 2 [file Table_1.docx]

**Multimedia Appendix 1.** Search Strategies for Each Database

**Pubmed:** ( "home mechanical ventilation" OR "domiciliary mechanical ventilation" OR "home ventilation" OR "long-term mechanical ventilation" OR "prolonged mechanical ventilation" OR (("home care" OR "home setting" OR "community setting" OR domiciliary OR home) AND ("mechanical ventilation" OR "ventilation, mechanical" OR "noninvasive ventilation" OR "non-invasive ventilation" OR NIV OR BiPAP OR "bilevel positive airway pressure" OR "tracheostomy ventilation")) ) AND (attitudes OR perceptions OR perspective OR perspectives OR experience OR experiences OR feelings OR needs OR demand OR support OR "support needs" OR barriers OR facilitators) AND (qualitative OR "focus group*" OR interview* OR experienc* OR attitud* OR feel* OR respons* OR perspectiv* OR opin* OR phenomenolog* OR "lived experience*" OR narrative* OR ethnograph* OR "grounded theory" OR "content analysis") AND 2020/01/01:2026/04/30[dp]

**WOS:** TS=(( ( "home mechanical ventilation" OR "domiciliary mechanical ventilation" OR "home ventilation" OR "long-term mechanical ventilation" OR "prolonged mechanical ventilation" OR ( ("home care" OR "home setting" OR "community setting" OR domiciliary OR home) AND ("mechanical ventilation" OR "ventilation, mechanical" OR "noninvasive ventilation" OR "non-invasive ventilation" OR NIV OR BiPAP OR "bilevel positive airway pressure" OR "tracheostomy ventilation") ) ) AND (attitudes OR perceptions OR perspective OR perspectives OR experience OR experiences OR feelings OR needs OR demand OR support OR "support needs" OR barriers OR facilitators) AND (qualitative OR "focus group*" OR interview* OR experienc* OR attitud* OR feel* OR respons* OR perspectiv* OR opin* OR phenomenolog* OR "lived experience*" OR narrative* OR ethnograph* OR "grounded theory" OR "content analysis") ))

**Embase:** ( 'home mechanical ventilation':ti,ab,kw OR 'domiciliary mechanical ventilation':ti,ab,kw OR 'home ventilation':ti,ab,kw OR 'domiciliary ventilation':ti,ab,kw OR 'long-term mechanical ventilation':ti,ab,kw OR 'long term mechanical ventilation':ti,ab,kw OR 'prolonged mechanical ventilation':ti,ab,kw OR 'home noninvasive ventilation':ti,ab,kw OR 'home non-invasive ventilation':ti,ab,kw OR 'domiciliary noninvasive ventilation':ti,ab,kw OR 'domiciliary non-invasive ventilation':ti,ab,kw OR 'home niv':ti,ab,kw OR 'domiciliary niv':ti,ab,kw OR 'bilevel positive airway pressure':ti,ab,kw OR 'home bipap':ti,ab,kw OR 'tracheostomy ventilation':ti,ab,kw OR ( ('home care':ti,ab,kw OR 'home setting':ti,ab,kw OR 'home-based':ti,ab,kw OR 'community setting':ti,ab,kw OR domiciliary:ti,ab,kw) AND ('mechanical ventilation':ti,ab,kw OR 'ventilation, mechanical':ti,ab,kw OR 'noninvasive ventilation':ti,ab,kw OR 'non-invasive ventilation':ti,ab,kw OR niv:ti,ab,kw OR bipap:ti,ab,kw OR 'bilevel positive airway pressure':ti,ab,kw OR 'tracheostomy ventilation':ti,ab,kw) ) ) AND ( experience*:ti,ab,kw OR perception*:ti,ab,kw OR perspective*:ti,ab,kw OR attitude*:ti,ab,kw OR feeling*:ti,ab,kw OR 'support need*':ti,ab,kw OR 'care need*':ti,ab,kw OR 'unmet need*':ti,ab,kw OR barrier*:ti,ab,kw OR facilitator*:ti,ab,kw ) AND ( qualitative:ti,ab,kw OR interview*:ti,ab,kw OR 'focus group*':ti,ab,kw OR phenomenolog*:ti,ab,kw OR ethnograph*:ti,ab,kw OR 'grounded theory':ti,ab,kw OR 'content analysis':ti,ab,kw OR 'thematic analysis':ti,ab,kw OR 'qualitative research'/exp ) AND [2020-2026]/py

## Cochrane: "home mechanical ventilation" OR "domiciliary mechanical ventilation" OR "home ventilation" OR "domiciliary ventilation" OR "long-term mechanical ventilation" OR "long term mechanical ventilation" OR "prolonged mechanical ventilation" OR "home noninvasive ventilation" OR "home non-invasive ventilation" OR "domiciliary noninvasive ventilation" OR "domiciliary non-invasive ventilation" OR "home NIV" OR "domiciliary NIV" OR "bilevel positive airway pressure" OR "home BiPAP" OR "tracheostomy ventilation" in Abstract OR "home care" OR "home setting" OR "home-based" OR "community setting" OR domiciliary in Abstract AND "mechanical ventilation" OR "ventilation, mechanical" OR "noninvasive ventilation" OR "non-invasive ventilation" OR NIV OR BiPAP OR "bilevel positive airway pressure" OR "tracheostomy ventilation" in Abstract AND experience* OR perception* OR perspective* OR attitude* OR feeling* OR "support need*" OR "care need*" OR "unmet need*" OR barrier* OR facilitator* in Abstract AND qualitative OR interview* OR "focus group*" OR phenomenolog* OR ethnograph* OR "grounded theory" OR "content analysis" OR "thematic analysis" in Abstract

**CINAHL：**( AB ("home mechanical ventilation" OR "domiciliary mechanical ventilation" OR "home ventilation" OR "domiciliary ventilation" OR "long-term mechanical ventilation" OR "long term mechanical ventilation" OR "prolonged mechanical ventilation" OR "home noninvasive ventilation" OR "home non-invasive ventilation" OR "domiciliary noninvasive ventilation" OR "domiciliary non-invasive ventilation" OR "home NIV" OR "domiciliary NIV" OR "bilevel positive airway pressure" OR "home BiPAP" OR "tracheostomy ventilation") OR (AB ("home care" OR "home setting" OR "home-based" OR "community setting" OR domiciliary OR home) AND AB ("mechanical ventilation" OR "ventilation, mechanical" OR "noninvasive ventilation" OR "non-invasive ventilation" OR NIV OR BiPAP OR "bilevel positive airway pressure" OR "tracheostomy ventilation"))) AND AB (experience* OR perception* OR perspective* OR attitude* OR feeling* OR "support need*" OR "care need*" OR "unmet need*" OR need* OR barrier* OR facilitator* OR caregiver* OR carer* OR "family caregiver*" OR "informal caregiver*" OR famil*) AND AB (qualitative OR interview* OR "focus group*" OR phenomenolog* OR ethnograph* OR "grounded theory" OR "content analysis" OR "thematic analysis" OR narrative*)
