# Supplementary material for: Experiences and support needs of patients receiving home mechanical ventilation and their caregivers: a qualitative meta-synthesis
Source: Front Public Health. 2026 Jul 2;14:1793552. doi: 10.3389/fpubh.2026.1793552 (PMC13373040; doi:10.3389/fpubh.2026.1793552)
Supplement: Supplementary file 3 [file Table_2.docx]

# Multimedia Appendix 2. Detailed Characteristics of Included Studies

| Author, year | Country | Sample | Study design | Aim | Data collection method | Data collection mode | Interview duration | Data analysis | Main findings/themes |
| --- | --- | --- | --- | --- | --- | --- | --- | --- | --- |
| Choyce et al. 2025  [29] | UK | 9 adults with cystic fibrosis using long-term domiciliary NIV | Descriptive qualitative study | To explore lived experiences of long-term domiciliary NIV | In-depth semi-structured interviews | Face-to-face, hospital setting | Average  1 h | Thematic analysis | **1.Gratitude**:   1. Ability to continue to live a fulfilling life; 2. Relationships and communication with the MDT; 3. Physical benefits of NIV;   d. Relationship with NIV.  2. **Determination despite challenges**:  a. Determination to accept treatment despite frightening preconceptions;  b. Determination to continue with NIV despite unpleasant side effects/drawbacks. |
| Esmaeili et al. 2022  [15] | Iran | 15 participants: 9 family caregivers, 3 home nurses, and 3 home care attendants | Qualitative study using conventional content analysis | To explore family caregivers’ needs in caring for adults receiving invasive HMV | Semi-structured interviews, structured observations, and field notes | Face-to-face, participant-selected settings | 30–90 min | Conventional content analysis | 1.Home caregivers  a.Educational needs (main educational needs and emergencyrelated needs),  b. Economic needs,  c.Psychological needs.  2.Caregivers  a.Pre-discharge preparation  b.Initial transition from hospital to home  c.Long-term follow-up. |
| Author, year | Country | Sample | Study design | Aim | Data collection method | Data collection mode | Interview duration | Data analysis | Main findings/themes |
| Ewers et al. 2022  [11] | Germany | 27 patients receiving HMV and 9 relatives | Explorative qualitative cross-sectional study | To explore users’ views on medical technical aid supply in HMV | Semi-structured interviews | Face-to-face or written, home/community settings | Average 45 min; range 14–105 min | Qualitative content analysis | 1.The journey of ventilation use begins: a. Being in an exceptional existential situation;  b. Being dependent, distracted, and unable to learn.  2. A bumpy start, but onwards we go:  a. Trying to adjust to ventilation, technology, and life at home;  b. Feeling challenged by collaboration with care providers.  3. The complex daily routine with HMV:  a. Gradually building trust in the technology;  b. Feeling the need to be constantly on guard.  4. The struggle to stay safe:  a. Dealing with risks, incidents, and complications;  b. Being left with many unanswered questions. |
| Israelsson-Skogsberg et al. 2024  [30] | Sweden | 9 young adults using HMV, aged 18–31 years; 2 received invasive ventilation via tracheostomy, 6 used NIV, and 1 used CPAP via facemask | Qualitative study using a phenomenological-hermeneutical methodology | To describe young adults’ everyday life with HMV | Narrative interviews | Home or online | 36–80 min | Phenomenological-hermeneutical analysis | 1.An everyday life with HMV is a walk on a line between independence and dependence: a. Being supported by medical technology; b. Personal care assistance—a vulnerable but necessary precondition for life; c. Owning an apartment—a possibility for freedom, but might be difficult to achieve. 2. An everyday life with HMV means being encountered in various ways: a. To be responded to as a unique person means to feel safe and capable; b. To confront prejudice; c. To be prepared to defend and argue for legal rights; d. To be extra vulnerable to external influencing factors—but not perceive illness. |
| Author, year | Country | Sample | Study design | Aim | Data collection method | Data collection mode | Interview duration | Data analysis | Main findings/themes |
| Khankeh et al. 2022  [31] | Iran | 28 participants: 14 professional healthcare workers,  12 family members, and 2 improved patients | Grounded theory study using the Strauss and Corbin approach | To explore the home health care process for mechanical ventilation-dependent patients | In-depth semi-structured interviews, supplemented by documents, field notes, nurses’ notes, news, and internet resources | Face-to-face, participant-selected settings | 16–72 min | Constant comparative analysis | **1.Challenging situation with stress and ambivalence**: a. Financial disruption; b. Chaotic family; c. Family emotional cohesion; d. Religious beliefs. 2. **Step-by-step care delegation**: a. Step-by-step care; b. Training of non-professional caregivers. 3. **Professional but limited**: a. Professional development of the nurse; b. Care in a complex and unfavorable context |
| Klingshirn et al. 2022  [32] | Germany | Qualitative component: 13 ventilated individuals and 18 family caregivers | Convergent parallel mixed-methods study | To compare care quality in private homes and shared living communities for long-term ventilated individuals | Semi-structured interviews | Face-to-face, written, or telephone | Ventilated individuals: mean 25 min, range 10–65 min; family caregivers: mean 28 min, range 13–55 min | Framework analysis; mixed-methods integration | **1.Fast access to reliable health advice**: 2. **Effective treatment delivered by trusted professionals**  **3.Continuity of care and smooth transitions**  4. **Involvement in decisions and respect for preferences**  5. **Clear information, communication, and support for self-care**  6. **Involvement of, and support for, family and carers**  7. **Emotional support, empathy, and respect**:  8. **Attention to physical and environmental needs** |
| Author, year | Country | Sample | Study design | Aim | Data collection method | Data collection mode | Interview duration | Data analysis | Main findings/themes |
| Mansell et al. 2020  [33] | UK | 34 participants: 15 patients, 5 carers, and 12 healthcare professionals | Qualitative study | To explore experiences of modem technology in home NIV management | Semi-structured interviews with patients and carers; focus groups with healthcare professionals | Telephone or face-to-face interviews; face-to-face focus groups | Not reported | Modified framework analysis | 1. **Surveillance: a paradox of findings**. 2. **Sanctions**. 3. **Complacency and ethics**. 4. **Efficiencies of healthcare provision**. 5. **Confidence: can be improved with technology**. |
| Perry et al. 2023  [34] | New Zealand | 11 people with neuromuscular disorders using NIV for more than 12 months; 3 caregivers attended interviews at the invitation of participants | Qualitative study guided by critical realism and contextualism | To explore healthcare experiences and system factors affecting NIV access and use | Semi-structured individual interviews | Home or online | Average 60 min | Reflexive thematic analysis | **1.Uptake of NIV therapy**: a. Informed consent; b. Establishing a clinical need for NIV; c. Establishing an effective therapeutic NIV setting. 2. **Practicalities of NIV**: a. Equipment set-up and care instructions; b. Getting the right machine and mask; c. Maintenance, servicing and upgrading of equipment; d. Resource allocation. 3. **Patient–clinician relationships**. |
| Thorborg et al. 2023  [35] | Denmark | 7 patients with ALS receiving non-invasive HMV and facing decisions about invasive HMV | Qualitative study using a phenomenological-hermeneutic approach | To explore ALS patients’ experiences and needs in decision-making about invasive HMV | Semi-structured interviews | Hospital or home | 30–90 min | Phenomenological-hermeneutic analysis | 1. **Being taken care of directly after receiving the diagnosis**. 2. **Living in uncertainty about what the future would bring**. 3. **Doubt causing patients with ALS to change their minds**. |
| Author, year | Country | Sample | Study design | Aim | Data collection method | Data collection mode | Interview duration | Data analysis | Main findings/themes |
| Wasilewski et al. 2022  [23] | Canada | 13 participants: 8 family caregivers of ventilator-assisted individuals living at home and 5 peer mentors | Qualitative descriptive study | To explore caregivers’ experiences with a web-based peer support program | Weekly online peer-to-peer group chat transcripts | Online text chats | Weekly chats over 9 weeks, approximately 1 h each | Thematic and framework analysis | **1.The experience of caregivers is characterized by unique challenges related to the complexity of VAI care including technology**: a. Hypervigilance is required; b. Boundary-setting is difficult; c. Role overload ensues. 2. **Mentors and caregiver participants reciprocally share support**: a. Assistance in applying disease management in daily life; b. Emotional and social support; c. Need for ongoing support. 3. **Despite hardships, there are things that make caregiving easier and joyful**: a. Tangible and emotional support from others; b. Compassionate care; c. Positive outlook on the caregiving journey. |
| Wilson et al. 2024  [36] | UK | 62 participants: 16 people living with MND using HMV, 10 family members, and 36 bereaved family members | Qualitative interview study using an interpretive constructivist methodology | To explore end-of-life decision-making about HMV use in MND | Flexible semi-structured interviews | Video, telephone, email, or face-to-face | Not reported | Thematic analysis with constant comparison | 1. **Selective decisions**. 2. **Timely decisions**. 3. **Defaulted decisions**. 4. **Proactive decisions**. |
| Winther et al. 2020  [37] | Denmark | 11 close relatives of people with ALS living at home with HMV or invasive HMV and formal caregivers | Qualitative study using a phenomenological-hermeneutic approach inspired by Ricoeur | To explore everyday life experiences of relatives of people with ALS receiving home mechanical ventilation | Semi-structured individual interviews and one focus group interview | Home, respiratory centre, or neutral setting | 32–60 min | Phenomenological-hermeneutic analysis | **1.We are in this together until the end**.2. **Vulnerable relatives fighting to keep track of everything**. 3. **Formal caregivers—a distressing relief**. 4. **A prison without personal space**. |
| Author, year | Country | Sample | Study design | Aim | Data collection method | Data collection mode | Interview duration | Data analysis | Main findings/themes |
| Yacob Hussain et al. 2025  [38] | Singapore | 20 people with chronic hypercapnic respiratory failure using home NIV for at least 6 months | Descriptive qualitative study guided by Roy’s adaptation model | To explore experiences of living with home NIV in chronic hypercapnic respiratory failure | One-time, audio-recorded face-to-face interviews | Face-to-face, sleep and assisted ventilation centre | 25 min | Thematic analysis | 1. **Forced acceptance to use NIV**. 2. **Experiencing symptoms relief and side effects**. 3. **Learning to maintain the NIV mask**. 4. **Incorporating NIV into the home environment**. 5. **Readjusting travelling activities**. 6. **NIV as part of their lives**. |

Note: For studies involving mixed home respiratory therapy populations, only qualitative findings clearly related to HMV users, HMV-related caregiving, or HMV-related care contexts were extracted. Oxygen therapy alone was not treated as HMV.
