# Supplementary material for: Experiences and support needs of patients receiving home mechanical ventilation and their caregivers: a qualitative meta-synthesis
Source: Front Public Health. 2026 Jul 2;14:1793552. doi: 10.3389/fpubh.2026.1793552 (PMC13373040; doi:10.3389/fpubh.2026.1793552)
Supplement: Supplementary file 4 [file Table_3.docx]

**Multimedia Appendix 3.** Correspondence Between Synthesized Themes, Subthemes, and Included Studies

| Synthesized themes | Subthemes | Corresponding Included Studies |
| --- | --- | --- |
| 1. Passive Entry, Repeated Weighing, and Active Participation in HMV Decision Making | a. Passive Acceptance and Insufficient Information at the Initial Stage | Choyce et al. [29]; Ewers et al. [11]; Perry et al. [34]; Thorborg et al. [35]; Yacob Hussain et al. [38] |
|  | b. Uncertainty and Repeated Weighing During Disease Progression | Thorborg et al. [35]; Wilson et al. [36]; Winther et al. [37] |
|  | c. From Reliance on Professional Judgment to Active Participation in Care Decisions | Khankeh et al. [31]; Perry et al. [34]; Wilson et al. [36]; Yacob Hussain et al. [38] |
|  | d. Choices Among Ventilation Modality, Disease Progression, and Family Caregiving Capacity | Choyce et al. [29]; Perry et al. [34]; Thorborg et al. [35]; Wilson et al. [36]; Khankeh et al. [31] |
| 2. Adapting to the Integration of Ventilation Technology into Everyday Family Life | a. Physical Discomfort, Operational Difficulties, and Emotional Fluctuations During Initial Adjustment | Choyce et al. [29]; Ewers et al. [11]; Perry et al. [34]; Yacob Hussain et al. [38] |
|  | b. Coexistence of Symptom Improvement and Device Related Adverse Effects | Choyce et al. [29]; Israelsson-Skogsberg et al. [30]; Yacob Hussain et al. [38] |
|  | c. Rearrangement of the Home Environment and Daily Rhythms | Esmaeili et al. [15]; Ewers et al. [11]; Khankeh et al. [31]; Wasilewski et al. [23]; Winther et al. [37]; Yacob Hussain et al. [38] |
|  | d. Balancing a Sense of Security, Remote Monitoring, and Privacy Ethics | Ewers et al. [11]; Mansell et al. [33]; Wasilewski et al. [23]; Winther et al. [37] |
| 3. Ongoing Tensions Among Life Support, Quality of Life, and Autonomy | a. Maintaining an Autonomous Life Under Technological Dependence | Choyce et al. [29]; Israelsson-Skogsberg et al. [30]; Klingshirn et al. [32]; Yacob Hussain et al. [38] |
|  | b. Stigma, Misunderstanding, and Defensiveness in Social Participation | Israelsson-Skogsberg et al. [30]; Klingshirn et al. [32]; Yacob Hussain et al. [38] |
|  | c. Coexistence of Improved Quality of Life and Restricted Living | Choyce et al. [29]; Esmaeili et al. [15]; Israelsson-Skogsberg et al. [30]; Klingshirn et al. [32]; Wasilewski et al. [23]; Yacob Hussain et al. [38] |
|  |  |  |
| Synthesized themes | Subthemes | Corresponding Included Studies |
|  | d. Sense of Control at the End of Life, Family Responsibility, and Decisions About Ventilation Withdrawal | Thorborg et al. [35]; Wilson et al. [36]; Winther et al. [37] |
| 4. Expansion of Family Caregiving Responsibilities and Reconstruction of the Boundaries of Professional Care | a. Transformation of Family Members Into Informal Caregivers With Technical Responsibilities | Esmaeili et al. [15]; Khankeh et al. [31]; Wasilewski et al. [23]; Winther et al. [37] |
|  | b. Formal Caregivers as Both a Source of Relief and Pressure Within the Home | Klingshirn et al. [32]; Winther et al. [37]; Wasilewski et al. [23] |
|  | c. Families Assuming the Role of System Coordinators | Ewers et al. [11]; Khankeh et al. [31]; Perry et al. [34]; Wasilewski et al. [23]; Winther et al. [37] |
|  | d. Care Setting and Professional Support Conditions Shaping Family Burden | Esmaeili et al. [15]; Klingshirn et al. [32]; Mansell et al. [33]; Perry et al. [34]; Winther et al. [37] |
| 5. Gaps in Support Systems and the Need for Continuous Support for the Whole Family | a. Insufficient Discharge Preparation and Continuing Education | Esmaeili et al. [15]; Ewers et al. [11]; Khankeh et al. [31]; Perry et al. [34]; Wasilewski et al. [23] |
|  | b. Insufficient Psychological Support and Peer Support | Esmaeili et al. [15]; Khankeh et al. [31]; Thorborg et al. [35]; Wasilewski et al. [23]; Wilson et al. [36]; Winther et al. [37] |
|  | c. Financial Burden and Insufficient Insurance Coverage | Esmaeili et al. [15]; Khankeh et al. [31]; Perry et al. [34]; Wasilewski et al. [23] |
|  | d. Difficulties in Service Accessibility, Equipment Supply, and System Navigation | Choyce et al. [29]; Ewers et al. [11]; Khankeh et al. [31]; Mansell et al. [33]; Perry et al. [34]; Wasilewski et al. [23] |
|  | e. Shifting the Target of Support From the Individual Patient to the Whole Family | Esmaeili et al. [15]; Ewers et al. [11]; Israelsson-Skogsberg et al. [30]; Klingshirn et al. [32]; Mansell et al. [33]; Perry et al. [34]; Wasilewski et al. [23]; Wilson et al. [36]; Winther et al. [37]; Yacob Hussain et al. [38] |
