# Supplementary material for: Experiences and support needs of patients receiving home mechanical ventilation and their caregivers: a qualitative meta-synthesis
Source: Front Public Health. 2026 Jul 2;14:1793552. doi: 10.3389/fpubh.2026.1793552 (PMC13373040; doi:10.3389/fpubh.2026.1793552)
Supplement: Supplementary file 5 [file Table_4.docx]

**Multimedia Appendix 4**. Full ConQual Assessment of the Synthesized Findings

| Synthesized findings | Contributing studies | Dependability | Credibility | ConQual rating |
| --- | --- | --- | --- | --- |
| Theme 1. Passive Entry, Repeated Weighing, and Active Participation in HMV Decision Making | Choyce et al. [29]; Ewers et al. [11]; Khankeh et al. [31]; Perry et al. [34]; Thorborg et al. [35]; Wilson et al. [36]; Winther et al. [37]; Yacob Hussain et al. [38] | High | Moderate | Moderate |
| 1a. Passive Acceptance and Insufficient Information at the Initial Stage | Perry et al. [34]; Thorborg et al. [35] | High | High | High |
| 1b. Uncertainty and Repeated Weighing During Disease Progression | Thorborg et al. [35]; Wilson et al. [36] | High | Moderate | Moderate |
| 1c. From Reliance on Professional Judgment to Active Participation in Care Decisions | Wilson et al. [36]; Khankeh et al. [31] | High | Moderate | Moderate |
| 1d. Choices Among Ventilation Modality, Disease Progression, and Family Caregiving Capacity | Perry et al. [34]; Choyce et al. [29]; Thorborg et al. [35]; Wilson et al. [36] | High | Moderate | Moderate |
| Theme 2. Adapting to the Integration of Ventilation Technology into Everyday Family Life | Choyce et al. [29]; Esmaeili et al. [15]; Ewers et al. [11]; Israelsson Skogsberg et al. [30]; Khankeh et al. [31]; Mansell et al. [33]; Perry et al. [34]; Wasilewski et al. [23]; Winther et al. [37]; Yacob Hussain et al. [38] | High | Moderate | Moderate |
| 2a. Physical Discomfort, Operational Difficulties, and Emotional Fluctuations During Initial Adjustment | Yacob Hussain et al. [38]; Ewers et al. [11] | High | Moderate | Moderate |
|  |  |  |  |  |
| Synthesized findings | Contributing studies | Dependability | Credibility | ConQual rating |
| 2b. Coexistence of Symptom Improvement and Device Related Adverse Effects | Yacob Hussain et al. [38]; Choyce et al. [29] | High | High | High |
| 2c. Rearrangement of the Home Environment and Daily Rhythms | Yacob Hussain et al. [38]; Ewers et al. [11]; Choyce et al. [29] | High | High | High |
| 2d. Balancing a Sense of Security, Remote Monitoring, and Privacy Ethics | Mansell et al. [33] | High | Moderate | Moderate |
| Theme 3. Ongoing Tensions Among Life Support, Quality of Life, and Autonomy | Choyce et al. [29]; Esmaeili et al. [15]; Israelsson Skogsberg et al. [30]; Klingshirn et al. [32]; Thorborg et al. [35]; Wasilewski et al. [23]; Wilson et al. [36]; Winther et al. [37]; Yacob Hussain et al. [38] | High | Moderate | Moderate |
| 3a. Maintaining an Autonomous Life Under Technological Dependence | Israelsson-Skogsberg et al. [30] | High | Moderate | Moderate |
| 3b. Stigma, Misunderstanding, and Defensiveness in Social Participation | Israelsson-Skogsberg et al. [30] | High | Moderate | Moderate |
| 3c. Coexistence of Improved Quality of Life and Restricted Living | Choyce et al. [29]; Wilson et al. [36]; Yacob Hussain et al. [38] | High | Moderate | Moderate |
| 3d. Sense of Control at the End of Life, Family Responsibility, and Decisions About Ventilation Withdrawal | Wilson et al. [36]; Thorborg et al. [35] | High | Moderate | Moderate |
|  |  |  |  |  |
| Synthesized findings | Contributing studies | Dependability | Credibility | ConQual rating |
| Theme 4. Expansion of Family Caregiving Responsibilities and Reconstruction of the Boundaries of Professional Care | Esmaeili et al. [15]; Ewers et al. [11]; Khankeh et al. [31]; Klingshirn et al. [32]; Mansell et al. [33]; Perry et al. [34]; Wasilewski et al. [23]; Winther et al. [37] | High | Moderate | Moderate |
| 4a. Transformation of Family Members Into Informal Caregivers With Technical Responsibilities | Winther et al. [37]; Khankeh et al. [31]; Esmaeili et al. [15] | High | High | High |
| 4b. Formal Caregivers as Both a Source of Relief and Pressure Within the Home | Winther et al. [37]; Israelsson-Skogsberg et al. [30] | High | Moderate | Moderate |
| 4c. Families Assuming the Role of System Coordinators | Winther et al. [37]; Wasilewski et al. [23] | High | Moderate | Moderate |
| 4d. Care Setting and Professional Support Conditions Shaping Family Burden | Winther et al. [37]; Esmaeili et al. [15]; Wasilewski et al. [23] | High | Moderate | Moderate |
| Theme 5. Gaps in Support Systems and the Need for Continuous Support for the Whole Family | Choyce et al. [29]; Esmaeili et al. [15]; Ewers et al. [11]; Israelsson Skogsberg et al. [30]; Khankeh et al. [31]; Klingshirn et al. [32]; Mansell et al. [33]; Perry et al. [34]; Thorborg et al. [35]; Wasilewski et al. [23]; Wilson et al. [36]; Winther et al. [37]; Yacob Hussain et al. [38] | High | Moderate | Moderate |
|  |  |  |  |  |
| Synthesized findings | Contributing studies | Dependability | Credibility | ConQual rating |
| 5a. Insufficient Discharge Preparation and Continuing Education | Esmaeili et al. [15]; Ewers et al. [11] | High | Moderate | Moderate |
| 5b. Insufficient Psychological Support and Peer Support | Esmaeili et al. [15]; Wasilewski et al. [23] | High | Moderate | Moderate |
| 5c. Financial Burden and Insufficient Insurance Coverage | Esmaeili et al. [15]; Khankeh et al. [31] | High | High | High |
| 5d. Difficulties in Service Accessibility, Equipment Supply, and System Navigation | Wasilewski et al. [23]; Winther et al. [37]; Mansell et al. [33] | High | Moderate | Moderate |
| 5e. Shifting the Target of Support From the Individual Patient to the Whole Family | Wasilewski et al. [23]; Esmaeili et al. [15]; Winther et al. [37]; Ewers et al. [11] | High | Moderate | Moderate |

**Note:** Dependability was assessed according to the methodological quality of the contributing studies, particularly the congruity between the stated philosophical perspective, methodology, research questions, data collection, data analysis, interpretation of results, and conclusions. Credibility was assessed according to the degree of support between the original participant quotations and the synthesized findings. The contributing studies generally demonstrated adequate methodological quality, although several studies provided limited reporting on the researchers’ cultural or theoretical positioning, reflexivity, and the influence of the researchers on the research process. These reporting limitations were considered when judging dependability but did not substantially compromise the overall methodological coherence of the synthesized findings. Subthemes supported by direct, consistent, and unequivocal participant quotations, such as passive acceptance and insufficient information at the initial stage, coexistence of symptom relief and device-related side effects, reorganization of the home environment and daily routines, transformation of family members into informal professionalized caregivers, and financial burden and insufficient insurance coverage, were assigned High ConQual ratings. Subthemes that involved broader interpretive synthesis across disease types, ventilation modalities, care settings, remote monitoring, professional feedback, or family support systems were assigned Moderate ConQual ratings. Based on these considerations, the final ConQual ratings for the synthesized themes and subthemes ranged from Moderate to High.
